# Supplementary material for: Absolute risk-based versus individualized benefit approaches for determining statin eligibility in primary prevention of cardiovascular diseases in Chinese populations: A modeling study
Source: PLoS Med. 2025 Jul 22;22(7):e1004556. doi: 10.1371/journal.pmed.1004556 (PMC12282892; doi:10.1371/journal.pmed.1004556)
Supplement: S1 Text — (DOCX) [file pmed.1004556.s001.docx]

## S1 Text. Supplementary Methods

### Imputation for the missing values

Missing values were imputed using the Multiple Imputations by Chained Equation (MICE) method [1]. The variables imputed included total cholesterol (TC), low-density lipoprotein cholesterol (LDL-C), high-density lipoprotein cholesterol (HDL-C), systolic blood pressure (SBP), diastolic blood pressure (DBP), and current smoking status. Missing values for the variables in the present study ranged from 0.1% (for smoking history) to 2% (for SBP). Information on age, sampling weight, sex, hypertension, hypertension treatment, and dyslipidaemia were used in the multiple imputation process. Continuous variables (TC, LDL-C, HDL-C, SBP, and DBP) were imputed using predictive mean matching, while the categorical variable (current smoking) was imputed using logistic regression. Five imputed datasets were generated [1].

### The absolute risk-based approach

We used the 2019 World Health Organization (WHO) cardiovascular risk equations [2] to calculate the probability of fatal and non-fatal cardiovascular disease (CVD) (10-year absolute CVD risk) based on baseline data, including age, sex, smoking status, systolic blood pressure, and TC levels. Because the WHO equations tend to overestimate CVD risk in Chinese populations (by 59% in men and 72% in women) [3], we recalibrated them by dividing the CVD risk by 1.59 for men and 1.72 for women. A 10-year CVD risk threshold of 10% was used to define high-risk individuals in Chinese populations [4]. According to the American College of Cardiology (ACC)/American Heart Association (AHA) guidelines [5], we define intermediate, borderline, and low risk using the following thresholds: 7.5%-10%, 5%-7.5%, and less than 5%, respectively.

### The individualized benefit approach based on the Causal-Benefit model

**1. The Causal-Benefit model**

Initially proposed by researchers seeking to refine traditional risk evaluation methods, the Causal-Benefit model shifts the focus from merely estimating the short‐term risk of clinical events to integrating the potential therapeutic benefits derived from addressing the underlying causes of atherosclerosis [6]. Unlike conventional approaches that rely solely on an individual’s 10-year baseline risk profile, the Causal-Benefit model incorporates crucial biomarkers, specifically LDL-C and atherogenic apolipoprotein B (apoB) lipoproteins—which are now recognized as key causal factors in the disease’s pathogenesis [7].

The model is underpinned by the principle that treatment decisions should be informed by a rigorous, quantitative assessment of the potential benefit versus the risk associated with therapy [7]. It explicitly calculates the anticipated benefit from interventions, such as LDL-C or apoB lowering, by considering both an individual’s baseline risk and lipid levels.

The primary objective of the Causal-Benefit model is to broaden the spectrum of individuals who may benefit from early intervention, with a particular emphasis on younger patients who conventional risk-based strategies might overlook. The model establishes eligibility for statin therapy by ensuring that the intervention falls below a specific maximum individual number needed to treat (iNNT), corresponding to a minimum individual absolute risk reduction (iARR) threshold equivalent to that observed with the traditional absolute risk-based approach. Empirical applications of the Causal-Benefit model have been explored in comparative studies conducted within diverse populations in the United States [8], Scotland [9], and Brazil [10].

**2. The applications of the Causal-Benefit model in the present study**

In the present study, for the individualized benefit approach, statin eligibility was determined by the individualized treatment benefit (ITE). Based on the Causal-Benefit model [7], we defined the individualized treatment benefit as the iARR from LDL-C reduction. We used the methods of Thanassoulis et al. to estimate the iARR for each person, assuming the benefit is primarily via LDL-C reduction, incorporating an interaction with baseline risk [6]. The iARR was calculated by the absolute CVD risk without statin and the effect of statin treatment on CVD risk.

In the present study, we intend to apply this model as follows: the definition of individualized benefit and the estimation of individualized statin treatment effects will follow the same methodology as in the original Causal-Benefit model. However, there are some differences compared to the original model in the thresholds of iARR to determine the statin initiation. We would calibrate the iARR thresholds to achieve event prevention similar to that of the risk-based approach. This allowed for a direct comparison of population characteristics and efficiency (e.g., iARR/iNNT distributions) under budget constraints in low- and middle-income countries. We would also consider the original threshold definitions used in the Causal-Benefit model to ensure comparability across studies. Details of the estimation of the statin treatment effect, the calculation of iARR, and the thresholds to determine the statin eligibility under the individualized benefit approach were described as follows:

**(1) The statin treatment effect on CVD risk**

We assumed that the effect of statin therapy on CVD risk primarily operates through the reduction of LDL-C [6]. Therefore, the relative risk (RR) of CVD from statin therapy was determined by estimating the absolute reduction in LDL-C and the RR of CVD per unit (mmol/L) of LDL-C reduction. Moderate-intensity statin therapy was assumed to reduce LDL-C by approximately 40% [5]. The LDL-C reduction for each participant was calculated by multiplying their baseline LDL-C by the 40% decrease from moderate-intensity statin therapy (**Formula 1**).

$Units of LDL-C reduction=40\%\times baseline LDL-C$ (1)

To estimate the RR of CVD per unit (mmol/L) of LDL-C reduction, we applied methods developed by Thanassoulis *et al*. [6], similar to those used in the original PIE model (**Formula 2 and 3**) [8]. **Formula 2** assumes that the RR of CVD per unit (mmol/L) of LDL-C reduction (RR_Thanassoulis_) is associated with the individual’s baseline quantitative CVD risk, which is estimated using the 2019 WHO laboratory-based equations [2].

${ln(\mathrm{RR}}_{\mathrm{Thanassoulis}})=-0.10821+0.12346\times\ln\left( baseline quantitative CVD risk \right)$ (2)

$\mathrm{RR}_{\mathrm{Thanassoulis}}=exp({ln(\mathrm{RR}}_{\mathrm{Thanassoulis}}))$ (3)

Finally, the relative risk of CVD from statin therapy (RR_statin-on-CVD_) was calculated by **Formula 4**.

$\mathrm{RR}_{statin on CVD}=\mathrm{RR}_{\mathrm{Thanassoulis}}^(units of LDL-C reduction)$ (4)

**(2) The expected risk reduction of statin therapy for each individual**

The expected risk reduction of statin therapy was defined as the difference between the 10-year absolute CVD risk with and without statin treatment (Risk_statin_ and Risk_no-statin_, respectively). Risk_no-statin_ is the baseline 10-year CVD risk calculated using the 2019 WHO laboratory-based models [2]. Risk_statin_ was estimated by multiplying the relative risk of CVD from statin therapy by Risk_no-statin_ (**Formula 5**). The expected risk reduction of statin therapy was then calculated by subtracting Risk_statin_ from Risk_no-statin_ (**Formula 6**).

$\mathrm{Risk}_{\mathrm{statin}}=\mathrm{RR}_{statin on CVD}\times\mathrm{Risk}_{no-statin}$ (5)

$Expected risk reduction=\mathrm{Risk}_{no-statin}-\mathrm{Risk}_{\mathrm{statin}}$ (6)

**(3) The iARR thresholds of the individualized benefit approach**

We select the threshold for the individualized benefit approach based on two principles: comparable CVD event prevention vs. minimum benefit expansion. Based on the two thresholds of the absolute risk-based approach (high-risk/at least intermediate-risk), four corresponding benefit thresholds will be established: (i) the high-benefit, (ii) the moderate-benefit, (iii) a minimum benefit as the high-risk groups, and (iv) a minimum benefit as the intermediate- and high-risk groups. The high-benefit cut-off was defined as the risk reduction threshold corresponding to the number of CVD events prevented in the high-risk group under the absolute risk-based strategy. Similarly, the moderate-benefit cut-off was defined as the risk reduction threshold corresponding to the number of events prevented in the intermediate- or higher-risk groups. To align with the Causal-Benefit model of Thanassoulis et al. [6], the minimum benefit threshold was defined as an iARR threshold that would treat all the adults who could gain at least a minimum benefit (the maximum iNNT) as the absolute risk-based approach.

### Detailed methods to estimate the outcomes and measures

This study used the code of the Prevention Impact and Efficiency (PIE) mode provided by Pletcher *et al.* [8] to compare the absolute risk-based and the individualized benefit approach.

**1. The number and the proportion of statin eligibility**

The statin eligibility criteria determined the number of statin treatments under each approach, accounting for the sampling weight of the CHARLS study [11]. The statin intervention proportion under each strategy refers to the percentage of individuals in the total population who meet the statin treatment criteria defined by that strategy.

**2. The number of CVD events averted**

The number of CVD events averted in ten years was calculated by multiplying the population average absolute CVD risk reduction (compared with the no-statin treatment pattern) by the size of the target population (i.e., the overall Chinese adults represented by the study participants), as shown in **Formula 7**.

$CVD events averted =（\sum_{i}^{n} W_{i}）\times({Risk\_Statin}_{\mathrm{average}}-{Risk\_No\_Statin}_{\mathrm{average}})$ (7)

Under each statin-treatment approach, the average CVD risk for the population under the treatment pattern (Risk_Statin_average_) and the no-statin treatment pattern (Risk_No_Statin_average_) were calculated separately using **Formula 8**. Each individual in the sample has a sampling weight to represent a group within the target population (i.e., Chinese adults aged 40 to 80 years in 2015 who meet the study’s eligibility criteria). The individual’s risk is multiplied by their sampling weight to reflect the total risk for the population they represent. For example, if an individual’s risk is 5% and their sampling weight is eight, this corresponds to eight individuals in the population with a 5% 10-year risk of developing CVD. In **Formula 8**, *Risk_average_* refers to the weighted average 10-year CVD risk of the target population, where *i* refers to each study participant, *n* is the number of participants in our sample; *W*_i_ is the sampling weight of each participant; and *Risk_i_* is the individual’s 10-year CVD risk. For participants eligible for statin therapy under the strategies for statin eligibility in this study (the absolute risk-based and the individualized benefit approaches), *Risk_i_* in **Formula 8** becomes the post-intervention risk; for those not eligible, it remains the pre-intervention risk. Under the no-statin treatment pattern, Risk_i_ is the pre-intervention risk for each participant.

$\mathrm{Risk}_{\mathrm{average}} =\sum_{i}^{n} {(W}_{i}\times\mathrm{Risk}_{i})/\sum_{i}^{n} W_{i}$ （8）

**3. The average number needed to treat (NNT)**

The number needed to treat (NNT) over ten years to prevent one CVD event was estimated within the treated subset and is equal to the reciprocal of the average CVD risk reduction in this subset. For each strategy, the treated subset consisted of individuals eligible for statin therapy. For example, under the individualized benefit approach, the treated subset includes those whose baseline expected benefit meets or exceeds the specified threshold.

As previously mentioned, each participant has a sampling weight representing a group of individuals in the target population. We multiplied each individual's risk reduction by their sampling weight to obtain the total risk reduction for the group they represent. For instance, if a participant in the treated subset has a risk reduction of 3% from statin treatment and a sampling weight of eight, this corresponds to eight people in the target population experiencing a 3% reduction in CVD risk due to statin therapy.

Therefore, the average risk reduction in the treated subset of the population is calculated as the weighted average of the individual CVD risk reductions (**Formula 9**). In **Formula 9**, Risk_Reduction_average_ represents the average risk reduction of the treated subset in the target population; *i* refers to each study participant in the treated subset, *m* is the number of participants in this subset; *W_i_* is the sampling weight of each participant; and Risk_Reduction_i_ is the difference between Risk_no statin_ and Risk_statin_ for each participant.

${Risk\_Reduction}_{\mathrm{average}} =\sum_{i}^{m} {(W}_{i}\times{Risk\_Reduction}_{i})/\sum_{i}^{m} W_{i}$ （9）

The NNT is then calculated as the reciprocal of this average risk reduction (**Formula 10**):

$\mathrm{NNT}=1/{Risk\_Reduction}_{\mathrm{average}}$ （10）

**4. The individualized number needed to treat (iNNT)**

The individual NNT (iNNT) was derived from the inverse of an individual's ARR, representing the number needed to treat individuals with specific characteristics over a specified period (e.g., 10 years) to prevent one CVD event [12].

**5. The data inputs**

The data inputs of the present study were summarized in Table A, including micro-data from nationally representative survey from the CHARLS study [11]; the percent LDL-C reduction of moderate-intensity statin therapy, extracted from the ACC/AHA of Cardiology Cholesterol management guideline, which synthesis the value of percent of LDL-C reductions of different type of statins from multiple data sources [5]; and the effect on CVD per mmol/L of LDL-C reduction, methods developed by Thanassoulis et al. [6], using data from the Cholesterol Treatment Trialists' (CTT) Collaboration meta-analysis [13].

**Table A. Data inputs**

| **Item** | **Data source and reference** |
| --- | --- |
| Individual-level information used to estimate 10-year absolute CVD risk | Nationally representative data [11] |
| age |  |
| sex |  |
| smoking status |  |
| systolic blood pressure |  |
| total cholesterol |  |
| Individual-level LDL-C | Nationally representative data [11] |
| The percent LDL-C reduction from moderate-intensity statin therapy | The ACC/AHA Cardiology Cholesterol management guideline management guideline [5] |
| The effect on CVD per mmol/L of LDL-C reduction | CTT meta-analysis [13] |

CVD indicates cardiovascular diseases; LDL-C, low-density lipoprotein cholesterol; ACC, American College of Cardiology; AHA, American Heart Association; CTT, the Cholesterol Treatment Trialists’ Collaboration.

### *References*

1. Chevret S, Seaman S, Resche-Rigon M. Multiple imputation: a mature approach to dealing with missing data. Intensive Care Med. 2015;41(2):348-350. doi: 10.1007/s00134-014-3624-x.
2. WHO CVD Risk Chart Working Group. World Health Organization cardiovascular disease risk charts: revised models to estimate risk in 21 global regions. Lancet Glob Health. 2019;7(10):e1332-e1345. doi: 10.1016/S2214-109X(19)30318-3.
3. Li J, Liu F, Yang X, Cao J, Chen S, Chen J, et al. Validating World Health Organization cardiovascular disease risk charts and optimising risk assessment in China. Lancet Reg Health West Pac. 2021;8:100096. doi: 10.1016/j.lanwpc.2021.100096.
4. Yang XL, Chen JC, Li JX, Cao J, Lu XF, Liu FC, et al. Risk stratification of atherosclerotic cardiovascular disease in Chinese adults. Chronic Dis Transl Med. 2016;2(2):102-109. doi: 10.1016/j.cdtm.2016.10.001.
5. Grundy SM, Stone NJ, Bailey AL, Beam C, Birtcher KK, Blumenthal RS, et al. 2018 AHA/ACC/AACVPR/AAPA/ABC/ACPM/ADA/AGS/APhA/ASPC/NLA/PCNA Guideline on the Management of Blood Cholesterol: A Report of the American College of Cardiology/American Heart Association Task Force on Clinical Practice Guidelines. J Am Coll Cardiol. 2019;73(24):e285-e350. doi: 10.1016/j.jacc.2018.11.003.
6. Thanassoulis G, Williams K, Altobelli KK, Pencina MJ, Cannon CP, Sniderman AD. Individualised Statin Benefit for Determining Statin Eligibility in the Primary Prevention of Cardiovascular Disease. Circulation. 2016;133(16):1574-1581. doi: 10.1161/CIRCULATIONAHA.115.018383.
7. Kohli-Lynch C, Thanassoulis G, Pencina M, Sehayek D, Pencina K, Moran A, et al. The Causal-Benefit Model to Prevent Cardiovascular Events. JACC Adv. 2024;3(3):100825. doi: 10.1016/j.jacadv.2023.100825.
8. Pletcher MJ, Pignone M, Jarmul JA, Moran AE, Vittinghoff E, Newman T. Population Impact & Efficiency of Benefit-Targeted Versus Risk-Targeted Statin Prescribing for Primary Prevention of Cardiovascular Disease. J Am Heart Assoc. 2017;6(2). doi: 10.1161/JAHA.116.004316.
9. Kohli-Lynch CN, Lewsey J, Boyd KA, French DD, Jordan N, Moran AE, et al. Beyond 10-Year Risk: A Cost-Effectiveness Analysis of Statins for the Primary Prevention of Cardiovascular Disease. Circulation. 2022;145(17):1312-1323. doi: 10.1161/CIRCULATIONAHA.121.057631.
10. Cesena FHY, Laurinavicius AG, Valente VA, Conceição RD, Nasir K, Santos RD, et al. Statin Eligibility in Primary Prevention: From a Risk-Based Strategy to a Personalized Approach Based on the Predicted Benefit. Am J Cardiol. 2018;121(11):1315-1320. doi: 10.1016/j.amjcard.2018.02.011.
11. Zhao Y, Hu Y, Smith JP, Strauss J, Yang G. Cohort profile: the China Health and Retirement Longitudinal Study (CHARLS). Int J Epidemio. 2014;43(1):61-68. doi: 10.1093/ije/dys203.
12. van der Leeuw J, Ridker PM, van der Graaf Y, Visseren FL. Personalised cardiovascular disease prevention by applying individualised prediction of treatment effects. Eur Heart J. 2014;35(13):837-843. doi: 10.1093/eurheartj/ehu004.
13. Cholesterol Treatment Trialists’ (CTT) Collaboration; Baigent C, Blackwell L, Emberson J, Holland LE, Reith C, et al. Efficacy and safety of more intensive lowering of LDL cholesterol: a meta-analysis of data from 170,000 participants in 26 randomised trials. Lancet. 2010;376(9753):1670-81. doi: 10.1016/S0140-6736(10)61350-5.
