# Supplementary material for: Absolute risk-based versus individualized benefit approaches for determining statin eligibility in primary prevention of cardiovascular diseases in Chinese populations: A modeling study
Source: PLoS Med. 2025 Jul 22;22(7):e1004556. doi: 10.1371/journal.pmed.1004556 (PMC12282892; doi:10.1371/journal.pmed.1004556)
Supplement: S2 Text — (DOCX) [file pmed.1004556.s002.docx]

## S2 Text. Statistical Analysis Plan

**Statistical Analysis Plan**

May 14^th^, 2024

This study used observational data. While formal pre-registration of the SAP was not required, we present the original plan below for transparency. This study is part of the project of “Modelling the Impacts of Reducing Risk factORs for CardioVascular Disease (MIRROR-CVD)”.

1. **Title**

- Absolute risk-based versus risk reduction-based strategies for determining statin eligibility in primary prevention of cardiovascular diseases in Chinese populations

1. **Objective**

- To quantify the population-level differences in statin eligibility and predicted cardiovascular disease (CVD) events prevented between the absolute risk-based and risk reduction-based strategies;
- To evaluate how the risk reduction-based approach reclassifies statin eligibility, prioritizing individuals with greater expected benefit from low-density lipoprotein cholesterol (LDL-C) lowering;
- To explore the potential clinical implications of using a risk reduction-based approach to guide patient-clinician shared decision-making in primary prevention.

1. **Data**

- Publicly available data from the China Health and Retirement Longitudinal Study (CHARLS).
- Parameters for statin treatment effects will be adopted from published literature [1-2].

1. **Study design**

Observational comparative modeling study.

1. **Outcomes**

- The projected number of CVD events averted over ten years
- Relative CVD risk reduction (%)
- The number of adults eligible for statin therapy
- The number needed to treat (NNT) over ten years to prevent one CVD event: the average NNT and the maximum individual number needed to treat (iNNT). The iNNT will be derived from the inverse of an individual's ARR, represents the number of individuals needed to treat over a specified period (e.g., 10 years) to prevent one CVD event [3]. The maximum iNNT for each strategy was defined as the NNT corresponding to the individual with the highest iNNT within that strategy [4].

1. **Methods**

- The outcomes would be estimated by the Prevention Impact and Efficiency (PIE) model proposed by Pletcher *et al.* [4], with the no-statin treatment pattern as the reference.
- The 2019 World Health Organization (WHO) laboratory-based equations [5] will be applied to estimate the 10-year absolute CVD risk.
- This study will follow the Strengthening the Reporting of Observational Studies in Epidemiology (STROBE) reporting guideline [6].
- Missing values for variables used in the 2019 WHO laboratory-based equations [5] will be imputed using the multiple imputations by chained equations (MICE) method [7].
- The CHARLS sampling weights [8] will be applied to all analyses to ensure national representativeness
- All statistical analyses will be performed using Stata/MP version 16.0 (StataCorp LLC, College Station, TX, USA)

1. **Primary analyses**

**Strategies of eligibility for statin therapy**

1. Absolute risk-based strategy. In this scenario, we will use the 2019 WHO laboratory-based equations [5] to calculate the probability of fatal and non-fatal CVD (10-year absolute CVD risk) based on baseline data, including age, sex, smoking status, systolic blood pressure, and total cholesterol (TC) levels. Because the WHO equations tend to overestimate CVD risk in Chinese populations (by 59% in men and 72% in women) [9], we recalibrate them by dividing the CVD risk by 1.59 for men and 1.72 for women. Two values of threshold will be considered: A 10-year CVD risk threshold of 10% in Chinese populations;[10], a value of 7.5% in the ACC/AHA guideline [11]. According to the ACC/AHA guideline, we define intermediate, borderline, and low risk using the following thresholds: 7.5%-10%, 5%-7.5%, and less than 5%, respectively [11].
2. Risk reduction-based strategy. In this scenario, the expected iARR will be defined as the difference in the 10-year absolute CVD risk with and without statin treatment. We will use the methods proposed by Thanassoulis *et al*.[1], to estimate the expected iARR. The high-benefit cut-off was defined as the risk reduction threshold corresponding to the number of CVD events prevented in the high-risk group under the absolute risk-based strategy. Similarly, the moderate-benefit cut-off will be defined as the risk reduction threshold corresponding to the number of events prevented in the intermediate or higher-risk groups.

**The main statistics to be reported**

The projected number of CVD events averted over ten years, relative CVD risk reduction (%), the number of adults eligible for statin therapy, and the number needed to treat (NNT) over ten years to prevent one CVD event.

1. **Sensitivity analyses**

The one-way sensitivity analyses will consider the effect of statin on LDL-C reduction and the study sample:

- Assuming a lower statin effect (30%) on LDL-C reduction [2].
- Expanding the data source to include not only individuals with blood samples but also other individuals from the 2015 cross-sectional sample of the CHARLS.

1. **Proposed Main Tables & Figures**

Table 1 Impact and efficiency of different strategies for primary prevention with statin therapy

- Reporting the CVD events averted (in thousands), Relative CVD risk reduction (%), Projected adult statin eligible (in millions), Average NNT and Maximum iNNT of the absolute Risk-based strategy and the risk reduction-based strategies.

Table 2 Comparisons of the baseline characteristics of the discordance groups by different strategies

- To compare the baseline characteristic of those uniquely selected by the absolute risk-based strategy (high-risk) and the risk reduction-based strategies (high-benefit)

Figure 1 Conceptual diagram of different strategies for statin eligibility

- To illustrate the metric (absolute risk, expected benefit) and the threshold of the absolute risk-based strategy and the risk reduction-based strategies

Figure 2 Discordance in statin eligibility by different strategies with comparable CVD events averted

- To show the overlap in statin eligibility by the absolute risk-based strategy and the risk reduction-based strategies with similar CVD events averted

Figure 3 Switchover from absolute risk-based to risk reduction-based strategy

- A Sankey diagram depicted the flow of individuals from absolute risk categories (borderline, intermediate, and high risk) to risk reduction groups (low, moderate, and high benefit)

Figure 4 Patient example to illustrate statin eligibility guided by the absolute risk and the risk reduction

- Showing the influence of different strategies on statin treatment allocation of individual patient

Figure 5 Illustration of how the risk reduction-based strategy prioritizes individuals based on treatment benefits rather than absolute risk alone

- Showing how the risk reduction-based strategy efficiently in selecting individuals with higher expected benefit from statin treatment

1. **References**
2. Thanassoulis G, Williams K, Altobelli KK, Pencina MJ, Cannon CP, Sniderman AD. Individualised Statin Benefit for Determining Statin Eligibility in the Primary Prevention of Cardiovascular Disease. Circulation 2016;133:1574-1581. doi: 10.1161/CIRCULATIONAHA.115.018383.
3. Grundy SM, Stone NJ, Bailey AL, Beam C, Birtcher KK, Blumenthal RS, et al. 2018 AHA/ACC/AACVPR/AAPA/ABC/ACPM/ADA/AGS/APhA/ASPC/NLA/PCNA Guideline on the Management of Blood Cholesterol: A Report of the American College of Cardiology/American Heart Association Task Force on Clinical Practice Guidelines. J Am Coll Cardiol 2019;73:e285-e350. doi: 10.1016/j.jacc.2018.11.003.
4. van der Leeuw J, Ridker PM, van der Graaf Y, Visseren FL. Personalised cardiovascular disease prevention by applying individualised prediction of treatment effects. Eur Heart J 2014;35:837-843. doi: 10.1093/eurheartj/ehu004.
5. Pletcher MJ, Pignone M, Jarmul JA, Moran AE, Vittinghoff E, Newman T. Population Impact & Efficiency of Benefit-Targeted Versus Risk-Targeted Statin Prescribing for Primary Prevention of Cardiovascular Disease. J Am Heart Assoc 2017;6. doi: 10.1161/JAHA.116.004316.
6. WHO CVD Risk Chart Working Group. World Health Organization cardiovascular disease risk charts: revised models to estimate risk in 21 global regions. Lancet Glob Health 2019;7:e1332-e1345. doi: 10.1016/S2214-109X(19)30318-3.
7. von Elm E, Altman DG, Egger M, Pocock SJ, Gotzsche PC, Vandenbroucke JP, et al. The Strengthening the Reporting of Observational Studies in Epidemiology (STROBE) statement: guidelines for reporting observational studies. Lancet 2007;370:1453-1457. doi: 10.1016/S0140-6736(07)61602-X.
8. Chevret S, Seaman S, Resche-Rigon M. Multiple imputation: a mature approach to dealing with missing data. Intensive Care Med 2015;41:348-350. doi: 10.1007/s00134-014-3624-x.
9. Zhao Y, Hu Y, Smith JP, Strauss J, Yang G. Cohort profile: the China Health and Retirement Longitudinal Study (CHARLS). Int J Epidemiol 2014;43:61-68. doi: 10.1093/ije/dys203.
10. Li J, Liu F, Yang X, Cao J, Chen S, Chen J, et al. Validating World Health Organization cardiovascular disease risk charts and optimising risk assessment in China. Lancet Reg Health West Pac 2021;8:100096. doi: 10.1016/j.lanwpc.2021.100096.
11. Yang XL, Chen JC, Li JX, Cao J, Lu XF, Liu FC, et al. Risk stratification of atherosclerotic cardiovascular disease in Chinese adults. Chronic Dis Transl Med 2016;2:102-109. doi: 10.1016/j.cdtm.2016.10.001.
12. Arnett DK, Blumenthal RS, Albert MA, Buroker AB, Goldberger ZD, Hahn EJ, et al. 2019 ACC/AHA Guideline on the Primary Prevention of Cardiovascular Disease: A Report of the American College of Cardiology/American Heart Association Task Force on Clinical Practice Guidelines. J Am Coll Cardiol 2019;74:e177-e232. doi: 10.1016/j.jacc.2019.03.010.
