# Supplementary material for: Absolute risk-based versus individualized benefit approaches for determining statin eligibility in primary prevention of cardiovascular diseases in Chinese populations: A modeling study
Source: PLoS Med. 2025 Jul 22;22(7):e1004556. doi: 10.1371/journal.pmed.1004556 (PMC12282892; doi:10.1371/journal.pmed.1004556)
Supplement: S3 Text — (DOCX) [file pmed.1004556.s003.docx]

## S3 Text. Post-Peer Review Analytical Enhancements

During the revision process, we refined the terminology used to describe the two strategies, incorporated additional subgroups, and further clarified the study outcomes to be reported, along with the corresponding figures and tables.

1. **Terminology**

We standardized the term "risk reduction-based strategy" to "individualized benefit approach" throughout, clarifying its conceptual basis in the Causal-Benefit Model [1,2].

1. **Subgroup analysis**
2. Added analyses to compare strategies in younger adults aged 40 to 60.
3. Conducted post hoc analyses comparing NNTs across subgroups based on concordance/discordance between risk/benefit criteria and including the group added by the Causal-Benefit model's minimum iARR threshold (details in S1 Text and Fig 2).
4. **Main statistics to be reported**

We added population eligibility percentages to results. Removed relative CVD risk reduction (%) from primary reporting to focus on absolute events averted.

1. **Main Tables & Figures**

Revised table structures for clarity (separating results by risk threshold). Added results explicitly showing outcomes using the Causal-Benefit model's minimum iARR threshold principle [1,2], in addition to the results based on thresholds calibrated for similar event prevention. The main tables and figures are as follows:

Table 1. Statin eligibilities, prevented CVD events, and efficiency of the individualized benefit approach compared with treating high-risk group

Table 2. Statin eligibilities, prevented CVD events, and efficiency of the individualized benefit approach compared with treating high-risk group (adults aged 40 to 60 years)

Table 3. Comparisons of the baseline characteristics of the discordance groups by different strategies (aged 40 to 60 years)

Fig 1. Conceptual diagram of different approaches for statin eligibility

Fig 2. The treatment efficiency of subgroups with different benefits and risk levels

Fig 3. Discordance in statin eligibility by different approaches with comparable CVD events averted

Fig 4. Switchover from absolute risk-based to individualized benefit approach

Fig 5. Patient example to illustrate statin eligibility guided by the absolute risk and the individualized benefit

Fig 6. Illustration of how the individualized benefit approach prioritizes individuals based on treatment benefits rather than absolute risk alone

1. **References**

1. Thanassoulis G, Williams K, Altobelli KK, Pencina MJ, Cannon CP, Sniderman AD. Individualised Statin Benefit for Determining Statin Eligibility in the Primary Prevention of Cardiovascular Disease. Circulation. 2016;133(16):1574-1581. doi: 10.1161/CIRCULATIONAHA.115.018383.

2. Kohli-Lynch C, Thanassoulis G, Pencina M, Sehayek D, Pencina K, Moran A, et al. The Causal-Benefit Model to Prevent Cardiovascular Events. JACC Adv. 2024;3(3):100825. doi: 10.1016/j.jacadv.2023.100825.
