# Supplementary figures and images for: Absolute risk-based versus individualized benefit approaches for determining statin eligibility in primary prevention of cardiovascular diseases in Chinese populations: A modeling study
Source: PLoS Med. 2025 Jul 22;22(7):e1004556. doi: 10.1371/journal.pmed.1004556 (PMC12282892; doi:10.1371/journal.pmed.1004556)

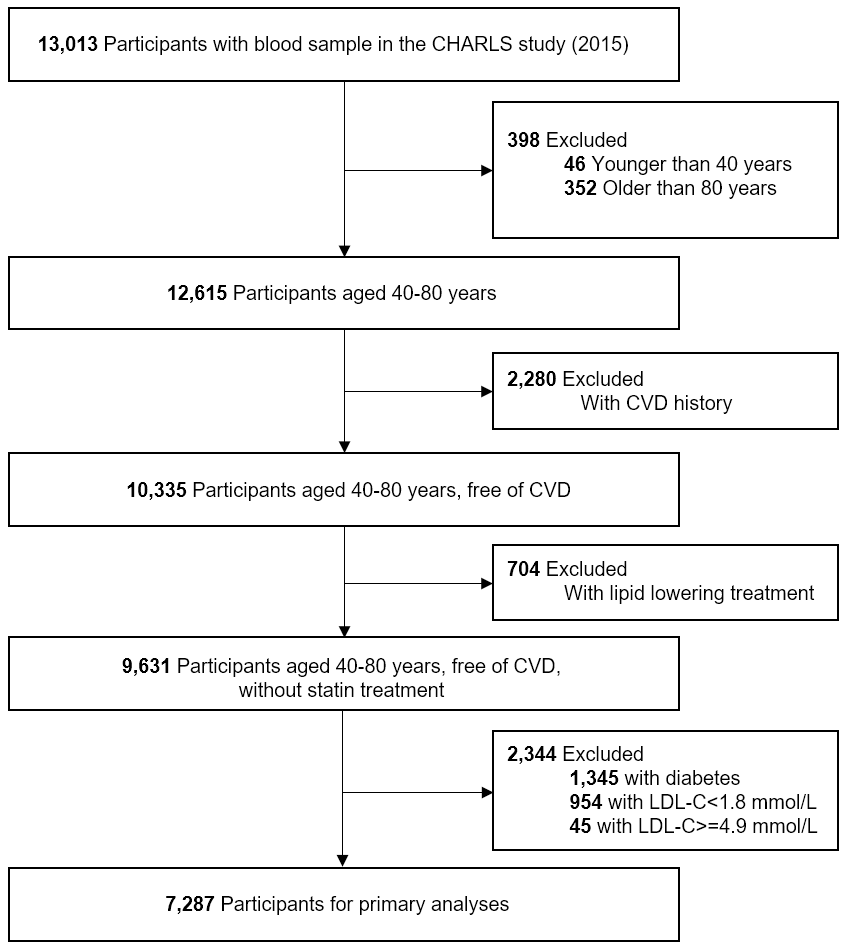

Supplement: S1 Fig — CHARLS indicates the China Health and Retirement Longitudinal Survey; CVD, cardiovascular disease, LDL-C, low-density lipoprotein cholesterol. (TIF) [file pmed.1004556.s005.tif]

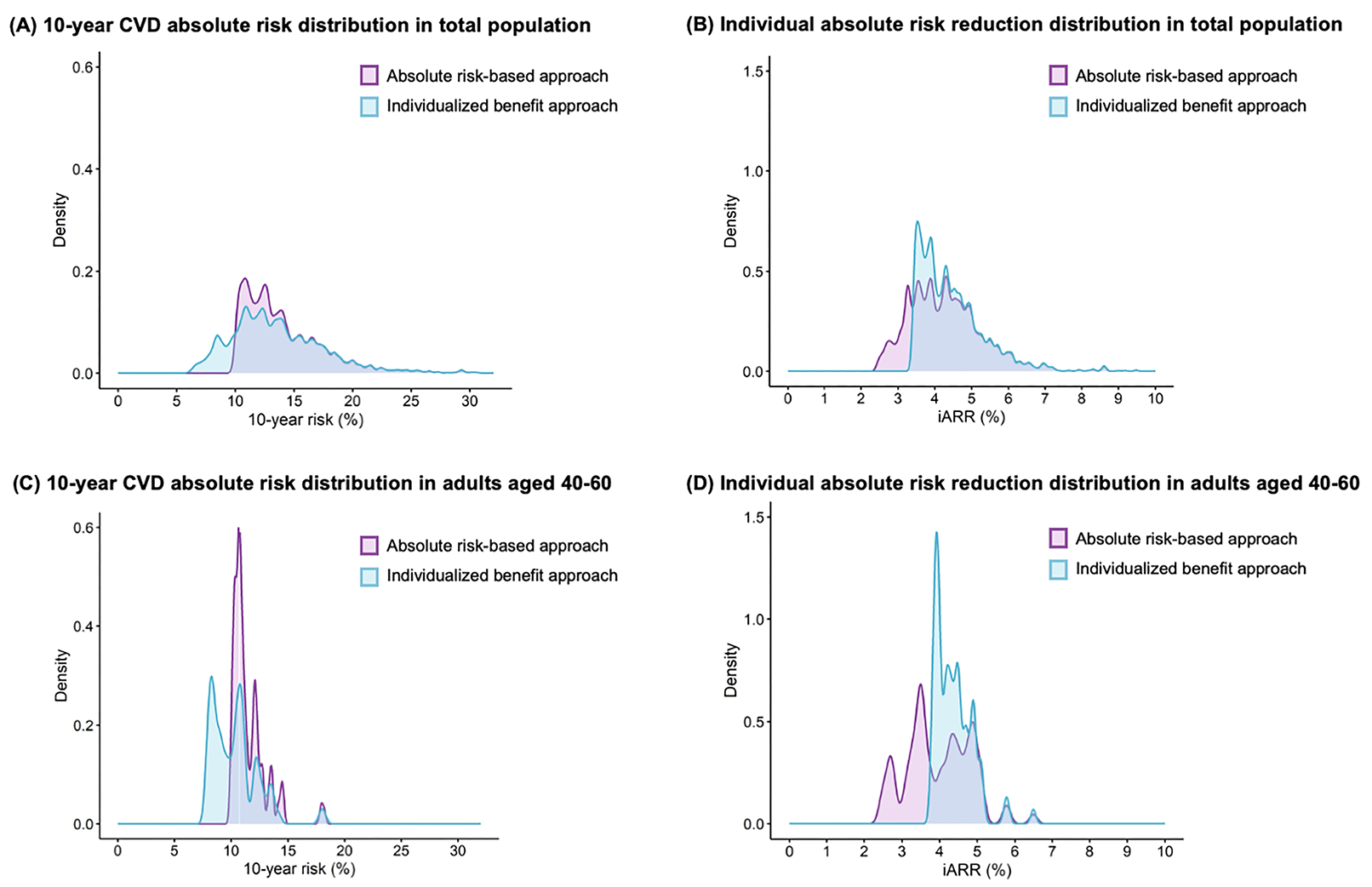

Supplement: S2 Fig — (A) In the total population, an iARR threshold of 3.4% in the total population, would avert a similar number of cardiovascular events to the absolute risk-based strategy when treating people in the high-risk group. (B) In the total population, an iARR threshold of 2.8% would avert a similar number of cardiovascular events to the absolute risk-based strategy approach when treating people in the intermediate- and high-risk groups. (C) For younger adults aged 40–60 years, an iARR threshold of 3.8% would avert a similar number of cardiovascular events to the absolute risk-based strategy when treating people in the high-risk group. (D) In adults aged 40–80 years, an iARR threshold of 3.0% would avert a similar number of cardiovascular events to the absolute risk-based strategy when treating people in intermediate- and high-risk groups. iARR indicates individual absolute risk reduction. (TIF) [file pmed.1004556.s006.tif]

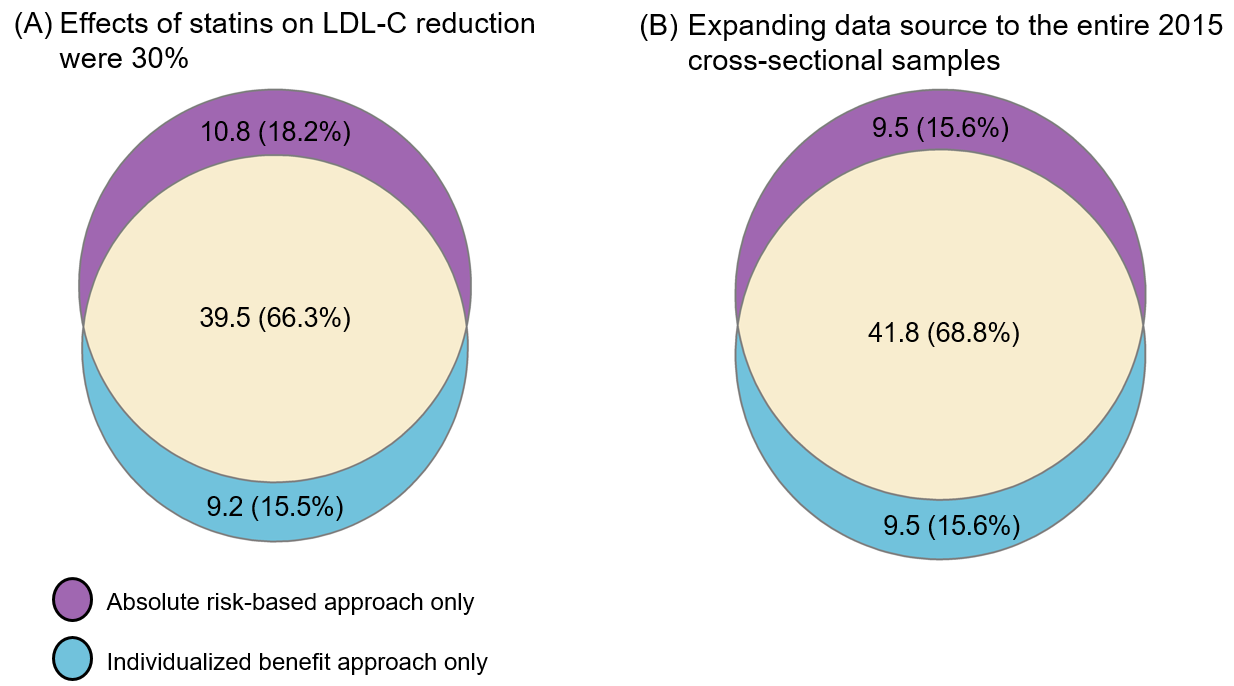

Supplement: S3 Fig — The absolute number of individuals eligible for statin therapy (in millions) and the corresponding percentages (%) were reported. (A) Under the individualized benefit approach, a 2.7% threshold would avert a similar number of CVD events as the absolute risk-based approach when treating people in high-risk group. (B) A 3.4% threshold of the individualized benefit approach would avert a similar number of CVD events as the absolute risk-based approach when treating people in high-risk group. CVD indicates cardiovascular disease, LDL-C, low-density lipoprotein cholesterol. (TIF) [file pmed.1004556.s007.tif]
