# Supplementary material for: Absolute risk-based versus individualized benefit approaches for determining statin eligibility in primary prevention of cardiovascular diseases in Chinese populations: A modeling study
Source: PLoS Med. 2025 Jul 22;22(7):e1004556. doi: 10.1371/journal.pmed.1004556 (PMC12282892; doi:10.1371/journal.pmed.1004556)
Supplement: S1 Table — Values are mean (SD) or % unless otherwise noted. aPresented as median (IQR). SBP indicates systolic blood pressure; DBP, diastolic blood pressure; TC, total cholesterol; LDL-C, low-density lipoprotein cholesterol; HDL-C, high-density lipoprotein cholesterol; IQR, interquartile range. (DOCX) [file pmed.1004556.s008.docx]

## S1 Table. Weighted characteristics of participants aged 40-80 years (main analysis)

| **Characteristics** | **All** | **Men** | **Women** |
| --- | --- | --- | --- |
| Unweighted No. | 7,287 | 3,399 | 3,888 |
| Weighted No. (millions) | 324.6 | 156.8 | 167.9 |
| Age (years) | 57.2 (9.4) | 58.0 (9.3) | 56.5 (9.5) |
| Current smoking | 28.6 | 54.8 | 4.1 |
| Hypertension | 33.1 | 36.0 | 30.3 |
| SBP (mmHg) | 126.0 (18.7) | 127.7 (18.4) | 124.4 (18.8) |
| DBP (mmHg) | 75.6 (11.7) | 77.1 (11.8) | 74.2 (11.5) |
| TC (mmol/L) | 4.9 (0.8) | 4.8 (0.8) | 5.0 (0.8) |
| LDL-C (mmol/L) | 2.8 (0.6) | 2.7 (0.6) | 2.8 (0.6) |
| HDL-C (mmol/L) | 1.4 (0.3) | 1.3 (0.3) | 1.4 (0.3) |
| Absolute risk (%)^a^ | 4.2 (2.3,7.4) | 5.6 (3.4,9.5) | 3.1 (1.8,5.4) |
| Absolute risk reduction (%)^a^ | 1.7 (1.1,2.7) | 2.1 (1.4,3.2) | 1.4 (0.8,2.2) |

Values are mean (SD) or % unless otherwise noted. ^a^Presented as median (IQR). SBP indicates systolic blood pressure; DBP, diastolic blood pressure; TC, total cholesterol; LDL-C, low-density lipoprotein cholesterol; HDL-C, high-density lipoprotein cholesterol; IQR, interquartile range.
