# Supplementary material for: Absolute risk-based versus individualized benefit approaches for determining statin eligibility in primary prevention of cardiovascular diseases in Chinese populations: A modeling study
Source: PLoS Med. 2025 Jul 22;22(7):e1004556. doi: 10.1371/journal.pmed.1004556 (PMC12282892; doi:10.1371/journal.pmed.1004556)
Supplement: S2 Table — Values are mean (SD) or % unless otherwise noted. aPresented as median (IQR). SBP indicates systolic blood pressure; DBP, diastolic blood pressure; TC, total cholesterol; LDL-C, low-density lipoprotein cholesterol; HDL-C, high-density lipoprotein cholesterol; IQR, interquartile range. (DOCX) [file pmed.1004556.s009.docx]

## S2 Table. Weighted characteristics of participants aged 40-80 years (expanding data source to the entire 2015 cross-sectional sample)

| **Characteristics** | **All** | **Men** | **Women** |
| --- | --- | --- | --- |
| Unweighted No. | 11,953 | 5,834 | 6,119 |
| Weighted No. (millions) | 346.3 | 168.2 | 178.1 |
| Age (years) | 57.2 (9.4) | 57.8 (9.2) | 56.7 (9.6) |
| Current smoking | 28.5 | 54.6 | 3.9 |
| Hypertension | 28.6 | 30.6 | 26.6 |
| SBP (mmHg) | 125.1 (18.3) | 126.7 (18.3) | 123.6 (18.2) |
| DBP (mmHg) | 75.1 (11.4) | 76.3 (11.5) | 73.9 (11.2) |
| TC (mmol/L) | 4.9 (0.8) | 4.8 (0.8) | 5.0 (0.8) |
| LDL-C (mmol/L) | 2.8 (0.6) | 2.7 (0.6) | 2.8 (0.6) |
| HDL-C (mmol/L) | 1.3 (0.3) | 1.3 (0.3) | 1.4 (0.3) |
| Absolute risk (%)^a^ | 4.1 (2.3,7.2) | 5.4 (3.3,9.0) | 3.0 (1.8,5.4) |
| Absolute reduction (%)^a^ | 1.7 (1.0,2.7) | 2.1 (1.4,3.1) | 1.4 (0.8,2.2) |

Values are mean (SD) or % unless otherwise noted. ^a^Presented as median (IQR). SBP indicates systolic blood pressure; DBP, diastolic blood pressure; TC, total cholesterol; LDL-C, low-density lipoprotein cholesterol; HDL-C, high-density lipoprotein cholesterol; IQR, interquartile range.
