# Supplementary material for: Absolute risk-based versus individualized benefit approaches for determining statin eligibility in primary prevention of cardiovascular diseases in Chinese populations: A modeling study
Source: PLoS Med. 2025 Jul 22;22(7):e1004556. doi: 10.1371/journal.pmed.1004556 (PMC12282892; doi:10.1371/journal.pmed.1004556)
Supplement: S3 Table — Point estimates and 95% CIs were reported, except the values of iARR were reported as median (the range from minimum to maximum). An iARR threshold of 2.8% would avert a similar number of CVD events to the absolute risk-based approach when treating people in the intermediate- and high-risk groups. An iARR of 2.0% is consistent with the minimum iARR of the intermediate- and high-risk groups. The CVD risk prediction was based on the 2019 World Health Organization laboratory-based equations incorporating age, sex, systolic blood pressure, total cholesterol, smoking status, and diabetes status [15]. Statin treatment effects were derived from the Cholesterol Treatment Trialists’ Collaboration meta-analysis [34], reflecting outcomes from multiple randomized controlled trials. CVD indicates cardiovascular diseases; NNT, number needed to treat; iARR, individual absolute risk reduction; CI, confidence interval. (DOCX) [file pmed.1004556.s010.docx]

## S3 Table. Statin eligibilities, prevented CVD events, and efficiency of the individualized benefit approach compared with treating intermediate- and high-risk groups

|  | **Absolute risk-based approach** |  | **Individualized benefit approach** | |
| --- | --- | --- | --- | --- |
|  | **Treat if at least intermediate risk (score>=7.5%)** |  | **Treat if at least moderate benefit (iARR>=2.8%)** | **Treat if gain at least a minimum benefit as the intermediate- and high-risk groups (iARR>=2.0%)** |
| **Population-level** |  |  |  |  |
| CVD events averted (in thousands) | 3041.4 (2847.5,3261.4) |  | 3046.3 (2847.0,3272.5) | 4460.2 (4245.6,4707.7) |
| Projected adult statin eligible (in millions) | 78.9 (73.9,83.8) |  | 76.7 (71.8,81.6) | 136.2 (129.9,142.5) |
| Proportion statin eligible (%) | 24.3 (22.8,25.8) |  | 23.6 (22.1,25.2) | 42.0 (40.0,43.9) |
| Average NNT | 26 (26,26) |  | 25 (25,26) | 31 (30,31) |
| **Individual-level** |  |  |  |  |
| iARR | 3.7 (2.0,9.5) |  | 3.7 (2.8,9.5) | 3.0 (2.0,9.5) |
| Maximum iNNT | 51 |  | 36 | 50 |

Point estimates and 95% CIs were reported, except the values of iARR were reported as median (the range from minimum to maximum). An iARR threshold of 2.8% would avert a similar number of CVD events to the absolute risk-based approach when treating people in the intermediate- and high-risk groups. An iARR of 2.0% is consistent with the minimum iARR of the intermediate- and high-risk groups. The CVD risk prediction was based on the 2019 World Health Organization laboratory-based equations incorporating age, sex, systolic blood pressure, total cholesterol, smoking status, and diabetes status [15]. Statin treatment effects were derived from the Cholesterol Treatment Trialists’ Collaboration meta-analysis [34], reflecting outcomes from multiple randomized controlled trials. CVD indicates cardiovascular diseases; NNT, number needed to treat; iARR, individual absolute risk reduction; CI, confidence interval.
