# Supplementary material for: Absolute risk-based versus individualized benefit approaches for determining statin eligibility in primary prevention of cardiovascular diseases in Chinese populations: A modeling study
Source: PLoS Med. 2025 Jul 22;22(7):e1004556. doi: 10.1371/journal.pmed.1004556 (PMC12282892; doi:10.1371/journal.pmed.1004556)
Supplement: S4 Table — Point estimates and 95% CIs were reported, except the values of iARR were reported as median (the range from minimum to maximum). An iARR threshold of 3.0% would avert a similar number of CVD events to the absolute risk-based strategy when treating people in the intermediate- and high-risk groups. An iARR of 2.0% is consistent with the minimum iARR of the intermediate- and high-risk groups. The CVD risk prediction was based on the 2019 World Health Organization laboratory-based equations incorporating age, sex, systolic blood pressure, total cholesterol, smoking status, and diabetes status [15]. Statin treatment effects were derived from the Cholesterol Treatment Trialists’ Collaboration meta-analysis [34], reflecting outcomes from multiple randomized controlled trials. CVD indicates cardiovascular diseases; NNT, number needed to treat; iARR, individual absolute risk reduction; CI, confidence interval. (DOCX) [file pmed.1004556.s011.docx]

## S4 Table. Statin eligibilities, prevented CVD events, and efficiency of the individualized benefit approach compared with treating intermediate- and high-risk groups (aged 40 to 60 years)

|  | **Absolute risk-based approach** |  | **Individualized benefit approach** | |
| --- | --- | --- | --- | --- |
|  | **Treat if at least intermediate risk (score>=7.5%)** |  | **Treat if at least moderate benefit (iARR>=3.0%)** | **Treat if gain at least a minimum benefit as the intermediate- and high-risk groups (iARR>=2.0%)** |
| **Population-level** |  |  |  |  |
| CVD events averted (in thousands) | 296.2 (244.3,351.8) |  | 305.2 (241.1,366.4) | 1005.3 (897.2,1124.4) |
| Projected adult statin eligible (in millions) | 8.8 (7.1,10.4) |  | 8.5 (6.8,10.3) | 38.0 (33.9,42.1) |
| Proportion statin eligible (%) | 4.2 (3.5,5.0) |  | 4.1 (3.3,5.0) | 18.1 (16.3,20.2) |
| Average NNT | 30 (29,31) |  | 28 (27,29) | 38 (37,39) |
| **Individual-level** |  |  |  |  |
| iARR | 3.3 (2.0,6.5) |  | 3.4 (3.0,6.5) | 2.5 (2.0,6.5) |
| Maximum iNNT | 50 |  | 33 | 50 |

Point estimates and 95% CIs were reported, except the values of iARR were reported as median (the range from minimum to maximum). An iARR threshold of 3.0% would avert a similar number of CVD events to the absolute risk-based strategy when treating people in the intermediate- and high-risk groups. An iARR of 2.0% is consistent with the minimum iARR of the intermediate- and high-risk groups. The CVD risk prediction was based on the 2019 World Health Organization laboratory-based equations incorporating age, sex, systolic blood pressure, total cholesterol, smoking status, and diabetes status [15]. Statin treatment effects were derived from the Cholesterol Treatment Trialists’ Collaboration meta-analysis [34], reflecting outcomes from multiple randomized controlled trials. CVD indicates cardiovascular diseases; NNT, number needed to treat; iARR, individual absolute risk reduction; CI, confidence interval.
