# Supplementary material for: Absolute risk-based versus individualized benefit approaches for determining statin eligibility in primary prevention of cardiovascular diseases in Chinese populations: A modeling study
Source: PLoS Med. 2025 Jul 22;22(7):e1004556. doi: 10.1371/journal.pmed.1004556 (PMC12282892; doi:10.1371/journal.pmed.1004556)
Supplement: S5 Table — Values are mean (SD) or %. SBP indicates systolic blood pressure; DBP, diastolic blood pressure; TC, total cholesterol; LDL-C, low-density lipoprotein cholesterol; HDL-C, high-density lipoprotein cholesterol. (DOCX) [file pmed.1004556.s012.docx]

## S5 Table. Comparisons of the baseline characteristics of the discordance groups by different strategies

| **Characteristics** | **Risk reduction ≥3.4% and**  **absolute risk <10%** |  | **Risk reduction <3.4% and**  **absolute risk ≥10%** | ***P* value** |
| --- | --- | --- | --- | --- |
| Unweighted No. | 212 |  | 225 | … |
| Weighted No. (millions) | 8.6 |  | 9.7 | … |
| Age (years) | 64.4 (6.7) |  | 69.1 (5.7) | <0.001 |
| men | 53.7 |  | 74.2 | 0.002 |
| Current smoking | 38.0 |  | 55.8 | 0.020 |
| Hypertension | 54.7 |  | 61.9 | 0.315 |
| SBP (mmHg) | 134.7 (18.6) |  | 138.0 (20.1) | 0.224 |
| DBP (mmHg) | 77.5 (11.1) |  | 77.0 (11.6) | 0.684 |
| TC (mmol/L) | 5.9 (0.7) |  | 4.2 (0.5) | <0.001 |
| LDL-C (mmol/L) | 3.7 (0.5) |  | 2.2 (0.2) | <0.001 |
| HDL-C (mmol/L) | 1.4 (0.3) |  | 1.3 (0.4) | 0.043 |

Values are mean (SD) or %. SBP indicates systolic blood pressure; DBP, diastolic blood pressure; TC, total cholesterol; LDL-C, low-density lipoprotein cholesterol; HDL-C, high-density lipoprotein cholesterol.
