# Supplementary material for: Absolute risk-based versus individualized benefit approaches for determining statin eligibility in primary prevention of cardiovascular diseases in Chinese populations: A modeling study
Source: PLoS Med. 2025 Jul 22;22(7):e1004556. doi: 10.1371/journal.pmed.1004556 (PMC12282892; doi:10.1371/journal.pmed.1004556)
Supplement: S6 Table — Point estimates and 95% CIs were reported, except the values of iARR were reported as median (the range from minimum to maximum). An iARR threshold of 2.7% would avert a similar number of CVD events to the absolute risk-based strategy when treating people in the high-risk group. An iARR of 2.0% is consistent with the minimum iARR of the high-risk group. The CVD risk prediction was based on the 2019 World Health Organization laboratory-based equations incorporating age, sex, systolic blood pressure, total cholesterol, smoking status, and diabetes status [15]. Statin treatment effects were derived from the Cholesterol Treatment Trialists’ Collaboration meta-analysis [34], reflecting outcomes from multiple randomized controlled trials. CVD indicates cardiovascular diseases; NNT, number needed to treat; iARR, individual absolute risk reduction; CI, confidence interval. (DOCX) [file pmed.1004556.s013.docx]

## S6 Table. Statin eligibilities, prevented CVD events, and efficiency of the individualized benefit approach compared with treating high-risk group (assuming a lower statin effect of 30% on LDL-C reduction)

|  | **Absolute risk-based approach** |  | **Individualized benefit approach** | |
| --- | --- | --- | --- | --- |
|  | **Treat if high risk (score>=10%)** |  | **Treat if high benefit (iARR>=2.7%)** | **Treat if gain at least a minimum benefit as the high-risk groups (iARR>=2.0%)** |
| **Population-level** |  |  |  |  |
| CVD events averted (in thousands) | 1691.4 (1548.3,1861.3) |  | 1709.1 (1576.3,1855.7) | 2788.5 (2620.1,2981.7) |
| Projected adult statin eligible (in millions) | 50.3 (46.0,54.6) |  | 48.7 (44.8,52.5) | 95.1 (89.5,100.8) |
| Proportion statin eligible (%) | 15.5 (14.2,16.9) |  | 15.0 (13.8,16.2) | 29.3 (27.6,31.1) |
| Average NNT | 30 (29,30) |  | 28 (28,29) | 34 (34,35) |
| **Individual-level** |  |  |  |  |
| iARR | 3.3 (1.9,7.5) |  | 3.4 (2.7,7.5) | 2.7 (2.0,7.5) |
| Maximum iNNT | 53 |  | 37 | 50 |

Point estimates and 95% CIs were reported, except the values of iARR were reported as median (the range from minimum to maximum). An iARR threshold of 2.7% would avert a similar number of CVD events to the absolute risk-based strategy when treating people in the high-risk group. An iARR of 2.0% is consistent with the minimum iARR of the high-risk group. The CVD risk prediction was based on the 2019 World Health Organization laboratory-based equations incorporating age, sex, systolic blood pressure, total cholesterol, smoking status, and diabetes status [15]. Statin treatment effects were derived from the Cholesterol Treatment Trialists’ Collaboration meta-analysis [34], reflecting outcomes from multiple randomized controlled trials. CVD indicates cardiovascular diseases; NNT, number needed to treat; iARR, individual absolute risk reduction; CI, confidence interval.
