# Supplementary material for: Absolute risk-based versus individualized benefit approaches for determining statin eligibility in primary prevention of cardiovascular diseases in Chinese populations: A modeling study
Source: PLoS Med. 2025 Jul 22;22(7):e1004556. doi: 10.1371/journal.pmed.1004556 (PMC12282892; doi:10.1371/journal.pmed.1004556)
Supplement: S7 Table — Point estimates and 95% CIs were reported, except the values of iARR were reported as median (the range from minimum to maximum). An iARR threshold of 2.2% would avert a similar number of CVD events to the absolute risk-based strategy when treating people in the intermediate- and high-risk groups. An iARR of 1.6% is consistent with the minimum iARR of the intermediate- and high-risk groups. The CVD risk prediction was based on the 2019 World Health Organization laboratory-based equations incorporating age, sex, systolic blood pressure, total cholesterol, smoking status, and diabetes status [15]. Statin treatment effects were derived from the Cholesterol Treatment Trialists’ Collaboration meta-analysis [34], reflecting outcomes from multiple randomized controlled trials. CVD indicates cardiovascular diseases; NNT, number needed to treat; iARR, individual absolute risk reduction; CI, confidence interval. (DOCX) [file pmed.1004556.s014.docx]

## S7 Table. Statin eligibilities, prevented CVD events, and efficiency of the individualized benefit approach compared with treating intermediate- and high-risk groups (assuming a lower statin effect of 30% on LDL-C reduction)

|  | **Absolute risk-based approach** |  | **Individualized benefit approach** | |
| --- | --- | --- | --- | --- |
|  | **Treat if at least intermediate risk (score>=7.5%)** |  | **Treat if at least moderate benefit (iARR>=2.2%)** | **Treat if gain at least a minimum benefit as the intermediate- and high-risk groups (iARR>=1.6%)** |
| **Population-level** |  |  |  |  |
| CVD events averted (in thousands) | 2387.0 (2234.4,2559.6) |  | 2457.3 (2294.4,2649.4) | 3505.9 (3337.3,3708.2) |
| Projected adult statin eligible (in millions) | 78.9 (73.9,83.8) |  | 79.3 (74.0,84.7) | 135.2 (128.9,141.4) |
| Proportion statin eligible (%) | 24.3 (22.8,25.8) |  | 24.4 (22.8,26.1) | 41.6 (39.7,43.6) |
| Average NNT | 33 (33,34) |  | 32 (32,33) | 39 (38,39) |
| **Individual-level** |  |  |  |  |
| iARR | 2.9 (1.5,7.5) |  | 2.9 (2.2,7.5) | 2.4 (1.6,7.5) |
| Maximum iNNT | 65 |  | 45 | 62 |

Point estimates and 95% CIs were reported, except the values of iARR were reported as median (the range from minimum to maximum). An iARR threshold of 2.2% would avert a similar number of CVD events to the absolute risk-based strategy when treating people in the intermediate- and high-risk groups. An iARR of 1.6% is consistent with the minimum iARR of the intermediate- and high-risk groups. The CVD risk prediction was based on the 2019 World Health Organization laboratory-based equations incorporating age, sex, systolic blood pressure, total cholesterol, smoking status, and diabetes status [15]. Statin treatment effects were derived from the Cholesterol Treatment Trialists’ Collaboration meta-analysis [34], reflecting outcomes from multiple randomized controlled trials. CVD indicates cardiovascular diseases; NNT, number needed to treat; iARR, individual absolute risk reduction; CI, confidence interval.
