# Supplementary material for: Absolute risk-based versus individualized benefit approaches for determining statin eligibility in primary prevention of cardiovascular diseases in Chinese populations: A modeling study
Source: PLoS Med. 2025 Jul 22;22(7):e1004556. doi: 10.1371/journal.pmed.1004556 (PMC12282892; doi:10.1371/journal.pmed.1004556)
Supplement: S8 Table — Point estimates and 95% CIs were reported, except the values of iARR were reported as median (the range from minimum to maximum). An iARR threshold of 3.4% would avert a similar number of CVD events to the absolute risk-based strategy when treating people in the high-risk group. An iARR of 2.5% is consistent with the minimum iARR of the high-risk group. The CVD risk prediction was based on the 2019 World Health Organization laboratory-based equations incorporating age, sex, systolic blood pressure, total cholesterol, smoking status, and diabetes status [15]. Statin treatment effects were derived from the Cholesterol Treatment Trialists’ Collaboration meta-analysis [34], reflecting outcomes from multiple randomized controlled trials. CVD indicates cardiovascular diseases; NNT, number needed to treat; iARR, individual absolute risk reduction; CI, confidence interval. (DOCX) [file pmed.1004556.s015.docx]

## S8 Table. Statin eligibilities, prevented CVD events, and efficiency of the individualized benefit approach compared with treating high-risk group (expanding data source to the entire 2015 cross-sectional sample)

|  | **Absolute risk-based approach** |  | **Individualized benefit approach** | |
| --- | --- | --- | --- | --- |
|  | **Treat if high risk (score>=10%)** |  | **Treat if high benefit (iARR>=3.4%)** | **Treat if gain at least a minimum benefit as the high-risk groups (iARR>=2.5%)** |
| **Population-level** |  |  |  |  |
| CVD events averted (in thousands) | 2224.6 (2063.2,2372.2) |  | 2286.5 (2110.1,2461.7) | 3669.5 (3486.5,3854.9) |
| Projected adult statin eligible (in millions) | 51.3 (48.1,54.6) |  | 51.3 (47.7,54.9) | 98.7 (94.4,103.1) |
| Proportion statin eligible (%) | 14.8 (13.9,15.8) |  | 14.8 (13.8,15.9) | 28.5 (27.3,29.8) |
| Average NNT | 23 (23,23) |  | 22 (22,23) | 27 (27,27) |
| **Individual-level** |  |  |  |  |
| iARR | 4.2 (2.4,9.5) |  | 4.2 (3.4,9.5) | 3.5 (2.5,9.5) |
| Maximum iNNT | 41 |  | 29 | 40 |

Point estimates and 95% CIs were reported, except the values of iARR were reported as median (the range from minimum to maximum). An iARR threshold of 3.4% would avert a similar number of CVD events to the absolute risk-based strategy when treating people in the high-risk group. An iARR of 2.5% is consistent with the minimum iARR of the high-risk group. The CVD risk prediction was based on the 2019 World Health Organization laboratory-based equations incorporating age, sex, systolic blood pressure, total cholesterol, smoking status, and diabetes status [15]. Statin treatment effects were derived from the Cholesterol Treatment Trialists’ Collaboration meta-analysis [34], reflecting outcomes from multiple randomized controlled trials. CVD indicates cardiovascular diseases; NNT, number needed to treat; iARR, individual absolute risk reduction; CI, confidence interval.
