# Supplementary material for: Absolute risk-based versus individualized benefit approaches for determining statin eligibility in primary prevention of cardiovascular diseases in Chinese populations: A modeling study
Source: PLoS Med. 2025 Jul 22;22(7):e1004556. doi: 10.1371/journal.pmed.1004556 (PMC12282892; doi:10.1371/journal.pmed.1004556)
Supplement: S9 Table — Point estimates and 95% CIs were reported, except the values of iARR were reported as median (the range from minimum to maximum). An iARR threshold of 2.8% would avert a similar number of CVD events to the absolute risk-based strategy when treating people in the intermediate- and high-risk groups. An iARR of 2.0% is consistent with the minimum iARR of the intermediate- and high-risk groups. The CVD risk prediction was based on the 2019 World Health Organization laboratory-based equations incorporating age, sex, systolic blood pressure, total cholesterol, smoking status, and diabetes status [15]. Statin treatment effects were derived from the Cholesterol Treatment Trialists’ Collaboration meta-analysis [34], reflecting outcomes from multiple randomized controlled trials. CVD indicates cardiovascular diseases; NNT, number needed to treat; iARR, individual absolute risk reduction; CI, confidence interval. (DOCX) [file pmed.1004556.s016.docx]

## S9 Table. Statin eligibilities, prevented CVD events, and efficiency of the individualized benefit approach compared with treating intermediate and high-risk groups (expanding data source to the entire 2015 cross-sectional sample)

|  | **Absolute risk-based approach** |  | **Individualized benefit approach** | |
| --- | --- | --- | --- | --- |
|  | **Treat if at least intermediate risk (score>=7.5%)** |  | **Treat if at least moderate benefit (iARR>=2.8%)** | **Treat if gain at least a minimum benefit as the intermediate- and high-risk groups (iARR>=2.0%)** |
| **Population-level** |  |  |  |  |
| CVD events averted (in thousands) | 3160.3 (2988.8,3339.1) |  | 3159.8 (2985.1,3347.6) | 4576.6 (4382.4,4784.9) |
| Projected adult statin eligible (in millions) | 81.8 (77.7,85.9) |  | 79.5 (75.4,83.6) | 139.2 (134.4,144.0) |
| Proportion statin eligible (%) | 23.6 (22.5,24.8) |  | 22.9 (21.8,24.2) | 40.2 (38.8,41.6) |
| Average NNT | 26 (26,26) |  | 25 (25,26) | 30 (30,31) |
| **Individual-level** |  |  |  |  |
| iARR | 3.7 (2.0,9.5) |  | 3.7 (2.8,9.5) | 3.0 (2.0,9.5) |
| Maximum iNNT | 51 |  | 36 | 50 |

Point estimates and 95% CIs were reported, except the values of iARR were reported as median (the range from minimum to maximum). An iARR threshold of 2.8% would avert a similar number of CVD events to the absolute risk-based strategy when treating people in the intermediate- and high-risk groups. An iARR of 2.0% is consistent with the minimum iARR of the intermediate- and high-risk groups. The CVD risk prediction was based on the 2019 World Health Organization laboratory-based equations incorporating age, sex, systolic blood pressure, total cholesterol, smoking status, and diabetes status [15]. Statin treatment effects were derived from the Cholesterol Treatment Trialists’ Collaboration meta-analysis [34], reflecting outcomes from multiple randomized controlled trials. CVD indicates cardiovascular diseases; NNT, number needed to treat; iARR, individual absolute risk reduction; CI, confidence interval.
