# Supplementary material for: Absolute risk-based versus individualized benefit approaches for determining statin eligibility in primary prevention of cardiovascular diseases in Chinese populations: A modeling study
Source: PLoS Med. 2025 Jul 22;22(7):e1004556. doi: 10.1371/journal.pmed.1004556 (PMC12282892; doi:10.1371/journal.pmed.1004556)
Supplement: Attachment — Submitted filename: Response_to_reviewers.docx [file pmed.1004556.s019.docx]

Point-by-point Response to comments

Manuscript no.: PMEDICINE-D-25-00419

**Reviewer reports:**

**Reviewer #1: This study re-visit the question on whether use of statin should be based on absolute risk of CVD or estimated absolute risk reduction. This is an interesting question given today's emphasis on precision prevention. The data and approach to the question appear to be robust overall. However, I have several comments:**

**Comment 1: The estimated number of CVD avoidable between two approaches were extremely close, with massive overlaps in 95% CIs (Table 1). The projected people eligible for statin and the average NNT were also very similar. The only discernible difference between the two methods are the maximum iNNT. However that statistic is only one person and I do wonder whether that warrants the study's strong conclusion 'offering enhanced efficiency than the absolute risk-based approach'. Also I wondered if iNNT makes sense - NNT is a measure for population, what does it tell to an individual?**

**Response:** We thank the Reviewer for acknowledging the robust nature of our data and approach and for raising these important points regarding the interpretation of our findings.

**(1) Similarity of population-level outcomes**: We agree the estimated events prevented, number eligible, and average NNT were very similar between the absolute risk-based and the risk reduction approaches (which we now term the 'individualized benefit approach' by the suggestion from the Reviewer#2). This similarity was by design, as we calibrated the benefit threshold to match event prevention for a controlled comparison. While this indicates similar population-level efficiency reflecting average treatment effects (ATE), our goal was also to consider the heterogeneity in individualized treatment effects (ITE), which might influence the medical treatment of individual patients.

**(2) Distinct populations and the role of iNNT**: Despite the similar overall impact, the two strategies identify substantially different groups of individuals for treatment (only 68.9% overlap between high-risk and high-benefit groups). This divergence motivated investigating efficiency at the individual level using metrics like the individual Number Needed to Treat (iNNT). We understand the Reviewer's query regarding this metric. Conceptually, iNNT estimates the NNT tailored to an individual's predicted absolute risk reduction (ARR), representing the number needed to treat with those specific characteristics to prevent one event over a defined period. This aligns with precision prevention goals by reflecting individual benefit potential. [*Eur Heart J*. 2014;35:837-43; *Circulation*. 2016;133:1574-1581; *J Am Heart Assoc*. 2017;6].

In the *Methods* section, we clarified the definition and rationale for using iNNT: "We also calculated individual NNT (iNNT=1/iARR) to assess individual-level efficiency. The iNNT represented the number needed to treat for individuals with specific characteristics over a specified period (e.g., 10 years) to prevent one CVD event [20]. The maximum iNNT within each strategy was noted, which was defined as the NNT corresponding to the individual with the highest iNNT within that strategy [19]." (Page2 11-12, Lines 125-130)

**(3) Comparing efficiency using iARR distribution**: We agree maximum iNNT is not robust. Following reviewer suggestions, we now focus on comparing the individual Absolute Risk Reduction (iARR) distributions. Our revised results show meaningful differences. While the risk-based group includes individuals with higher absolute risk, it also has a longer lower-end tail in the iARR distribution (**S2 Fig**), meaning it selects more individuals with low predicted benefit. Specifically, 19.3% (9.7 million) selected by the risk-based strategy had an iARR<3.4% (equivalent to iNNT>29). In contrast, none selected by the similarly calibrated individualized benefit approach had an iNNT>29. This demonstrates the individualized benefit approach's greater efficiency in excluding individuals with relatively lower benefit.

This finding is now added in the *Results* section: "Although the median iARR was similar between the high-risk and high-benefit groups (**Table 1**), the distribution for the iARR in high-risk group exhibited a longer lower end-tail compared to the high-benefit group (**S2B Fig**). This indicates the risk-based approach selects a larger proportion of individuals with low predicted benefit. Critically, 19.3% (9.7 million in 50.3 million) selected by the risk-based approach had iARR <3.4% (iNNT>29), whereas none selected by the benefit approach fell below this threshold (**S2B Fig**). The difference in iARR distribution tails was particularly evident in younger adults (aged 40-60) **(S2D Fig)**. This demonstrates the superior efficiency of the individualized benefit approach in excluding individuals predicted to derive minimal absolute benefit from statin therapy." (Page 17, Lines 255-266)

**(4) Additional evidence supporting efficiency**: Further analyses, prompted partly by Reviewer #2, provide additional support for the efficiency of the individualized benefit approach:

**(a) Subgroup analysis (Aged 40-60)**: In younger adults, the benefit approach achieved similar event prevention with a lower average NNT (23 vs. 25). The iARR/iNNT distributions also showed greater separation in this subgroup (**S2 Fig**), and the overlap between selected groups was only 35.5% (**Fig 3**). Individuals uniquely selected by the individualized benefit approach had lower blood pressure but higher LDL-C than those uniquely selected by the absolute risk-based approach (**Table 3**), suggesting better targeting towards lipid-driven risk and away from risk driven primarily by other factors like blood pressure. This supports the precision of the benefit approach, particularly in younger adults.

**(b) Alternative threshold scenario**: If we adopted an alternative approach as the causal-benefit model proposed by Reviewer #2, setting the iARR threshold such that the minimum benefit (maximum iNNT) in the high-benefit group matched that of the risk-based group (max iNNT=41, corresponding min iARR=2.5%), the individualized benefit approach would prevent significantly more CVD events (3.57 million *vs.* 2.16 million) while ensuring no one received treatment with less predicted benefit than the least-benefitting person under the current risk-based standard. This analysis is described in the *Results* section. (Page 15, Lines 197-202)

In summary, the distinct populations selected, favorable iARR distribution, lower NNT in younger adults, and greater event prevention under the alternative threshold collectively support the conclusion that the individualized benefit approach offers advantages in efficient targeting. We have revised the manuscript's conclusion to reflect this evidence, focusing on improved targeting and individual-level efficiency. Acknowledging the reviewer's point about the term 'enhanced efficiency', we have moderated the language in the Conclusion while maintaining that the efficiency advantage, particularly avoiding treatment for individuals with relatively lower benefit, is supported.

**Comment 2: perhaps a clearer approach to show the ARR approach to be better (for individuals) would be to examine the distribution of ARR. From the data shown (similar average NNT but different max iNNT), i'd expect the ARR would have a longer lower end tail in the absolute risk approach.**

**Response:** We appreciate this constructive suggestion. Examining the individual Absolute Risk Reduction (iARR) distributions directly addresses this point. Confirming the Reviewer's expectation, our analysis shows the absolute risk-based approach included a longer lower-end tail of iARR values compared to the individualized benefit approach (**S2 Fig**). This indicates the absolute risk-based approach selects more individuals with relatively low predicted absolute benefit (low iARR).

As suggested, we added a density plot (**S2 Fig**) and described the findings in the *Results* section as "Although the median iARR was similar between the high-risk and high-benefit groups (**Table 1**), the distribution for the iARR in high-risk group exhibited a longer lower end-tail compared to the high-benefit group (**S2B Fig**). This indicates the risk-based approach selects a larger proportion of individuals with low predicted benefit. Critically, 19.3% (9.7 million in 50.3 million) selected by the risk-based approach had iARR <3.4% (iNNT>29), whereas none selected by the benefit approach fell below this threshold (**S2B Fig**). The difference in iARR distribution tails was particularly evident in younger adults (aged 40-60) **(S2D Fig)**. This demonstrates the superior efficiency of the individualized benefit approach in excluding individuals predicted to derive minimal absolute benefit from statin therapy." (Page 17, Lines 255-266).

**Comment 3: Critically, this study assumes the effect of statin on CVD prevention is only via LDL-c reduction. I believe this assumption requires strong justification. This is a strong assumption that could overestimate the efficiency of ARR reduction approach, since the ARR is based on LDL-c level. There are studies that showed statin not only reduces circulatory LDL-c but also stabilises plagues (https://pubmed.ncbi.nlm.nih.gov/30642643/). Instead, I wondered why the effect of statin is not modelled based on clinical trials? There are trials that estimated CVD risk reduction directly.**

**Response:** We thank the Reviewer for raising this critical point regarding the mechanisms of statin benefit and our modeling assumptions.

**(1) The rationale for focusing on LDL-C reduction**: We acknowledge the Reviewer's point that statins exert beneficial effects beyond LDL-C lowering (e.g., plaque stabilization) [*Trends Cardiovasc* *Med*. 2019;29:451-455]. However, robust evidence, primarily from large meta-analyses of trials, establishes LDL-C reduction as the principal driver of statin-mediated CVD risk reduction, with risk reduction proportional to the magnitude of LDL-C lowering. [*Lancet*. 2010;376:1670-81; *Lancet*. 2016;388:2532-2561]

While clinical trials report average effects, estimating individualized benefit necessitates modeling interactions involving the primary mechanism. Average effects often assume constant proportional risk reduction, which may not hold for individuals. [*Nature Medicine*. 2024; 30, 958–968] Therefore, consistent with established methodologies for modeling statin treatment effects [*JAMA Cardiol*. 2019;4:969-977; *Circulation*. 2022;145:1312-1323; *JAMA*. 2024;332:989-1000] and the Causal-Benefit model framework [*JACC Adv*. 2024;3:100825; *Circulation*. 2016;133:1574-1581; *J Am Heart Assoc*. 2017;6], we focused on the LDL-C lowering pathway. While a simplification, this approach is a common and often necessary strategy in comparative modeling studies focusing on individual benefit prediction due to the complexities of modeling multiple pathways at an individual level.

Furthermore, in low- and middle-income countries (LMIC) settings with budget constraints and different risk factor profiles (e.g., high prevalence of hypertension), prioritizing individuals likely to benefit most from statins' primary mechanism (LDL-C lowering) is important. Our finding that the benefit approach better identifies younger individuals with higher LDL-C and excludes some high-risk individuals driven by non-lipid factors (e.g., high blood pressure) supports its potential efficiency in this context.

**(2) Addressing potential overestimation and limitation:** We agree this assumption is a key limitation and acknowledge it in the *Discussion*. Focusing solely on LDL-C could influence comparative efficiency estimates. The net impact (over- or under-estimation of relative efficiency) is uncertain, as it depends on the distribution of non-LDL mediated benefits (e.g., from plaque stabilization) among the distinct groups selected by each strategy. For instance, if high-ARR individuals also gain more from pleiotropic effects, we might underestimate their total benefit; conversely, if such effects are greater in high-risk individuals not selected by high ARR, the relative efficiency could shift. Assessing this is precluded by data limitations.

Given that neglecting non-LDL pathways might particularly underestimate benefits for high-risk individuals with lower LDL-C, an alternative, as applied in the Causal-Benefit model framework discussed with Reviewer #2, is to use a minimum iARR threshold derived from the risk-based group. This ensures all high-risk individuals are eligible while still identifying lower-risk, high-benefit individuals, potentially capturing broader benefits. This highlights the importance of context, resources, and shared decision-making in choosing a threshold strategy.

We added literature on pleiotropic effects [*Trends Cardiovasc* *Med*. 2019;29:451-455] and revised the limitations section (Page 23, Lines 390-404): "Second, consistent with prior modeling studies [10,19,32,33], we assumed quantifiable benefit stems primarily from LDL-C reduction. While LDL-C lowering is the principal mechanism for statin benefit [34,35], this simplification neglects other effects like plaque stabilization [36] and could underestimate benefits, particularly for high-risk individuals with lower LDL-C uniquely selected by the risk-based approach. An alternative, ensuring all high-risk individuals remain eligible, is applying the Causal-Benefit model's minimum iARR threshold [11]. This allows broader identification of potential beneficiaries (via LDL-C or other mechanisms) but requires adequate resources and supports shared decision-making regarding threshold choice."

**Reviewer #2: This analysis compares two approaches to select individuals to prevent ASCVD. One is the conventional model- the absolute risk approach; the second is the absolute risk reduction approach, which is based on the Causal-Benefit model to prevent ASCVD.1 The analysis was designed to compare the two approaches to prevent an equal number of events. Their results show the absolute risk approach was slightly superior. Unfortunately, their analysis does not reproduce the intent of the new model- the Causal Benefit model of cardiovascular prevention because it does not reproduce the methods of the new model. Indeed, the methods of their analysis frustrate the purpose for which it was developed. I will explain.**

**All the accepted models to select individuals for pharmacological treatment to prevent cardiovascular disease use a risk period of 10 years. In this regard, all follow the pattern of the original model- the Framingham model risk score. I presume Framingham picked a 10-year period to predict risk because this was, at the time, the longest period for which they had reliable data. But this singular decision- to limit the prediction of ASCVD risk to 10 years- has had multiple adverse consequences, which many still fail to appreciate.**

**Chief among these is that the non-modifiable factors that increase cardiovascular risk- age and sex- account for the overwhelming majority of risk whereas the modifiable causal factors- the apoB lipoproteins, blood pressure, diabetes- account for little of the risk.2 This means that age and sex become the determining factors for selection. Thus, for the American Guidelines, virtually all men over 60 are eligible, whereas a much smaller number of men and almost no women under 60 are eligible.3 This is an inappropriate result for multiple reasons, not least the biases against women and those who are younger.**

**More fundamentally, risk is not the same as incidence. Notwithstanding that the 10-year risk threshold is not commonly crossed until after age 60, about 40-45% of all ASCVD events occur before age 60.4 Why? If risk is so much lower in those under 60, how can the incidence of events be so close? The answer is simple. Risk is the number of events per standard number of individuals per unit time. Incidence is the total number of events. And there many more under 60 than over 60. Accordingly, larger numbers at lower risk produce large numbers with events. Furthermore, by any measure that matters, a premature heart attack or a premature stroke or a premature death or incapacity due to vascular disease is more tragic, more costly to the individual, their family and society than the same events in those who are older, who have had more opportunity to create and contribute. But there is yet another fatal flaw in the conventional model: many of the ASCVD events that occur after 60 are due to the progression of disease that developed before age 60 and continued to progress until after age 60, disease whose progression could have been prevented by proven medical therapy.**

**The risk model did make sense. Treat those most likely to have an event. But it is the time frame- the period of 10 years that converts the risk model from a strategy based on common sense to a strategy based on no pathophysiologic or epidemiologic sense. This is why we developed the Causal -Benefit model.1,5 Not to replace the Risk model. But to extend and amplify it. However, our model is not the model the authors present in this paper. The distinctions in purpose and method and outcomes are categorical.**

**In our model, we calculate the minimum Absolute Risk Reduction (ARR) in those who would be selected for pharmacological therapy by the conventional risk model. We then identify those amongst the general population, who would have a similar ARR but are below the threshold level of risk to be eligible for pharmacological therapy. These are added to the initial risk group. By design, therefore, the population selected by the ARR approach must be larger than the conventional approach and the levels of LDL-C (or whatever the determinative marker selected is- preferably apoB). We found that "Among 2013 guideline-eligible individuals, the mean 10-year predicted risk was 13.9% (range, 7.5%-43.5%) and the expected ARR10 from statins was 4.8% (range, 2.3%-10.6%), whereas for the individualized**  **statin benefit approach, the mean 10-year predicted risk was 10.9% (range, 3.7%-43.5%) and the expected ARR10 was 4.0% (range, 2.3%-10.6%). This corresponds to a group NNT10 of 21 (range, 9-44) and 25 (range, 9-44) for statin eligibility based on 2013 guidelines or individualized benefit-based approach, respectively." 4 Amongst those added in the lower risk but acceptable benefit group, the average age was less and a greater number were women. The LDL-C, of course was higher. 4**

**By contrast, the major findings in the present analysis are: "49.2 million (95%CI: 45.3-53.0) and 50.3 million (95%CI: 46.0-54.6) adults would be eligible for statins therapy under the risk reduction-based and absolute risk-based strategies, respectively. Among 58.9 million adults recommended for statin therapy by either strategy, 16.5% of individuals are uniquely identified by the absolute risk-based approach and 14.6% by the risk reduction-based approach, with only 68.9% overlap."**

**Positive features do emerge in the present paper: the ARR group is younger, has higher levels of LDL-C, and includes more females. But because the authors have committed themselves to a strategy that prevents an equal number of events with both strategies, they throw away the potential for gain. What is the logic for capping gain? Treating more with moderate risk is cost-effective?6 The outcome the authors have chosen is to cap is benefit for the people they have chosen to serve, not extend it. What is the argument against multiplying the benefit of preventing the most common cause of death and disability of humankind? In our view, further work must be done. We should move beyond the 10-year horizon of risk. Earlier intervention will produce greater benefit.7**

**I do not doubt the authors' good intentions. But the strategy they have chosen frustrates their good intentions. I do hope the authors reconsider the principles on which they have based their analysis. I cannot insist they change. But I do insist that their paper be revised so as to indicate why they reach one result, whereas we reach another, very different result. They have not applied our method and this is why they have not reproduced our results.**

**I disagree with how they have constructed their analysis and I have attempted to set out why. But my criticisms are offered in a spirit of collegiality and good will.**

**Response:** We are incredibly grateful to the Reviewer for the extensive, insightful feedback and the detailed explanation of the Causal-Benefit model's rationale. We agree the Causal-Benefit model improves on conventional risk-based approaches by considering individual benefit related to causal factors and using a minimum benefit threshold to expand eligibility, particularly for younger individuals. Indeed, the model's advantages and supporting evidence from high-income settings (e.g., the U.S. and Scotland) [*Circulation.* 2016;133:1574-1581; *J Am Heart Assoc*. 2017;6; *Circulation*. 2022;145:1312-1323] motivated our study applying these principles in China, an LMIC with different risk factor profiles. We aimed to evaluate the individualized benefit approach based on this model against the conventional approach in this context. We apologize if our initial description lacked clarity regarding our purpose and alignment with the Causal-Benefit framework [*Circulation*. 2016;133:1574-1581; *JACC Adv*. 2024;3:100825]. Below, we clarify this alignment, explain differences arising from study settings, and present new analyses as requested.

**(1) The methodological consistency**: Statin eligibility strategies involve a metric (e.g., absolute risk, individualized benefit/iARR) and a threshold (i.e., 7.5% for CVD risk, or 2.3% for individualized benefit). We argue our metric for the individualized benefit approach aligns with the Causal-Benefit model; the primary difference lies in the threshold choice, which we initially adapted based on the LMIC setting.

**(a) The metric used in the statin treatment strategies:** We compared the absolute risk-based approach (metric=10-year risk) with the individualized benefit approach (metric=iARR). Consistent with the Reviewer's work [*Circulation*. 2016;133:1574-1581], we estimated iARR assuming benefit is primarily via LDL-C reduction, incorporating an interaction with baseline risk. In this revision, we added text to the *Introduction* and *Methods* explicitly stating our individualized benefit approach is conceptually based on the Causal-Benefit model: "An individualized benefit approach, conceptually based on the Causal-Benefit model framework [10,11], formalizes this by evaluating the predicted individual absolute risk reduction (iARR) from LDL-C lowering." (Page 6, Line 16-19) and "The individualized benefit approach, conceptually based on the Causal-Benefit model [11], guided eligibility by predicted iARR from moderate-intensity statin therapy (**S1 Appendix**). Consistent with this framework and established methods for estimating individualized benefit [10,11], we assumed LDL-C reduction is the primary mediator of statin benefit for calculating iARR (details in **S1 Appendix**)." (Page 10, Line 86-91). In addition, based on the Reviewer's feedback and broader literature [*JAMA Cardiol*. 2017;2:1175-1176; *JACC Adv*. 2024;3:100825], we also adopted the "individualized benefit approach" terminology throughout.

**(b) The threshold to select the individuals recommended for statin use**: The Causal-Benefit model typically uses a minimum iARR threshold derived from the risk-based group to expand treatment. We acknowledge our initial analysis used a different threshold, calibrated to achieve similar event prevention as the risk-based approach. This allowed direct comparison of population characteristics and efficiency (e.g., iARR/iNNT distributions) under that constraint. The rationale was context-specific: in LMICs like China with low baseline statin use [*The Lancet Global health*. 2022;10:e369-e379] and different risk profiles (e.g., high hypertension burden) [*Eur Heart J*. 2022;43:2852-2863; *Lancet*. 2020;395:795-808], prioritizing efficient targeting within potential resource constraints is crucial.

**(2) The study objective and definition of "superiority"**: The differing thresholds reflect different primary objectives, influenced by setting. The Reviewer's statement that our results showed the risk approach as "slightly superior" misinterprets our conclusion; we found the benefit approach showed enhanced efficiency (better targeting, excluding very low-benefit individuals) when calibrated for equal events prevented. The Reviewer's application prioritizes expansion via a minimum benefit threshold. Both are valid objectives. We believe both studies demonstrate the advantages of considering individual benefit; ours provides an LMIC perspective on the Causal-Benefit model's efficiency and flexibility, informing its application in diverse contexts, as discussed in the manuscript: "In LMICs, where statin use is low despite significant eligibility, efficiently identifying suitable individuals is critical. Transitioning to an individualized benefit approach significantly alters the eligible population profile, potentially aligning better with priorities in resource-limited settings. Integrating predicted benefits into shared decision-making, supported by the Causal-Benefit model and other tools, offers a tailored approach [11]." (Page 21, Line 349-354).

**(3) New analysis applying the Reviewer's approach**: To directly address the Reviewer's core point and facilitate comparison, we applied their minimum benefit threshold principle (using min iARR=2.5% from the high-risk group in our population). Consistent with their model, this significantly expanded eligibility (96.3 vs. 50.3 million) and prevented more events (3.57 vs. 2.16 million) compared to the risk-based approach alone (**Table 1**). We also analyzed NNTs across subgroups defined by the different strategies (**Fig 2**). NNTs were lowest for those recommended by both (22), followed by benefit-only (26), then risk-only (33), and highest for the low-risk/low-ARR group added by the minimum benefit threshold (35). This highlights different efficiencies across subgroups, informing prioritization in resource-constrained settings.

These findings are now explicitly presented in the *Results* section: "Applying the Causal-Benefit model's minimum benefit threshold principle, using the minimum iARR observed in the high-risk group (iARR ≥2.5% in our population), the individualized benefit approach substantially expanded eligibility compared to the standard high-risk approach (29.7% [96.3 million] vs. 15.5% [50.3 million]) and consequently prevented significantly more events (3.57 million vs. 2.16 million) (Table 1). Analysis of NNT across subgroups defined by risk/benefit status revealed varying efficiency levels (Fig 2): NNT was lowest for individuals meeting both high-risk and high-benefit criteria (NNT=22), intermediate for those meeting only high-benefit (NNT=26) or only high-risk (NNT=33) criteria, and highest for the lower-risk/lower-benefit group added by applying the minimum iARR threshold (NNT=35). This suggests potential efficiency gradients for prioritization." (Page 15, Lines 197-209)

We also discussed in detail, highlighting the alignment with the Causal-Benefit model's potential when applied for expansion: "Applying the Causal-Benefit model's minimum benefit threshold (iARR≥2.5%) substantially increased events prevented (65%) by expanding eligibility, demonstrating the model's potential for enhancing overall prevention. However, our subgroup analysis revealed varying efficiency levels within this expanded group. Therefore, considering budget constraints in LMICs, healthcare context-specific iARR thresholds are crucial to balance maximal prevention benefits with efficient resource allocation." (Page 21, Lines 355-361)

We acknowledge the Reviewer's important point about moving beyond the 10-year risk horizon. While necessary for comparability, we agree lifetime perspectives are critical and added this limitation: "We modeled only 10-year benefits; longer-term models might show greater efficiency, especially for younger adults [28].". (Page 23, Line 401-403) In addition, as suggested by Reviewer #3, we added the Discussion of the advantages of the long-term benefit approach in the Discussion section. (Page 22, Line 372-374)

In summary, we hope these clarifications, new analyses, and revisions address the concerns raised. By comparing the risk-based strategy with the Causal-Benefit model applied using two different threshold approaches (equal event prevention vs. minimum benefit expansion) within an LMIC setting, we believe the paper now demonstrates the model's flexibility and advantages, offering evidence for its broader implementation. We deeply appreciate the Reviewer's engagement, which significantly improved the manuscript.

**Reviewer #3: General comments**

**The authors compare two strategies to determine statin eligibility in primary prevention: one traditional, based on the absolute calculated CVD risk, and the other based on the predicted absolute risk reduction with statin therapy. The authors claim with robust, convincing data that the second strategy is more efficient.
The manuscript is original and reports relevant results on statin eligibility in primary prevention. It is very well-written, high-quality text.
The methodology is complex, but a detailed explanation is provided in the supplement.**

**Response:** We sincerely thank the Reviewer #3 for the positive assessment of our manuscript's originality, relevance, methodological rigor, and overall quality. We appreciate the recognition of our findings regarding the efficiency of the individualized benefit approach.

**Specific comments
1. An important point that is not clear in the text is the** **percentage of the population that would be statin eligible by either strategy to prevent a similar number of events. I suggest that the authors mention this information, preferentially in both scenarios (treating high risk/high benefit and treating at least intermediate risk/at least moderate benefit).**

**Response:** We thank the Reviewer for pointing out the need for this clarification. As suggested, we have now explicitly stated the percentage of the total population eligible for statin therapy under the absolute risk-based strategy and risk reduction-based strategy (which we now term the "individualized benefit approach"), respectively, when calibrated to prevent a similar number of events. This information is provided for both comparison scenarios presented in the manuscript (i.e., high-risk vs. high-benefit groups and intermediate-or-higher risk vs. moderate-or-higher benefit groups). This information has been added to the Results section (Page 14, Lines 173-186).

**2. More importantly, in my opinion, is the shift of statin-eligible individuals to younger persons with fewer risk factors but higher LDL-c with the risk reduction-based strategy, compared to the traditional approach using the absolute calculated risk. This was also shown in previous studies addressing this topic (doi 10.1161/CIRCULATIONAHA.115.018383 [ref 10] and doi 10.1016/j.amjcard.2018.02.011). Authors may choose to mention that the observed results coincide with previous reports as a way of reinforcing the validity of this relevant finding.**

**Response:** We agree with the Reviewer that the observed shift in characteristics of eligible individuals toward younger age and higher LDL-C levels (despite fewer traditional risk factors) with the individualized benefit approach is a crucial finding. We also appreciate the suggestion to contextualize this with prior research. Accordingly, we have added a statement to the Discussion section acknowledging that our results align with previous studies, thereby reinforcing the validity of this observation. We have cited the specific studies noted by the Reviewer [*Circulation*. 2016;133:1574-1581; *Am J Cardiol*. 2018;121:1315-1320]. The added text is: "Our findings that the benefit approach uniquely identifies younger individuals, more women, and those with higher LDL-C align with prior studies in U.S. [10] and Brazilian [25] populations." (Page 22, Line 364-366)

**3. In the Discussion, the authors state "Early intervention in younger adults is critical, as cumulative LDL-C exposure during young and middle adulthood is associated with increased CVD risk, and the benefits of LDL-C lowering are more substantial with prolonged treatment." As a suggestion, I understand that this sentence would be a great opportunity to cite a paper by Thanassoulis et al (doi 10.1001/jamacardio.2018.3476) showing data in favor of a long-term benefit-based strategy for statin eligibility in younger persons.**

**Response:** We appreciate this helpful suggestion to cite relevant work on long-term benefit strategies. We agree that the paper by Thanassoulis et al. [JAMA Cardiol. 2018;3:1090-1095] provides important context for the Discussion on early intervention. As suggested, we have now cited this paper [JAMA Cardiol. 2018;3:1090-1095] in the Discussion section. The relevant text now includes: "Individualized benefit approaches incorporating long-term [28] or lifetime perspectives [29] may be particularly useful for guiding decisions in younger adults compared to standard 10-year models.". (Page 22, Line 372-374)

**4. Results from analyses considering statin therapy using the moderate-benefit cut-off or for intermediate-risk group are shown in Table 1 and Figure 2, but are not mentioned in the main text. I suggest that the authors comment on these findings in the text.**

**Response:** We thank the Reviewer for noting that the results for the intermediate-risk/moderate-benefit scenario, while presented in Table 1 (**S3 Table** in the new revision) and Figure 2 (**Fig 3** in the new revision), were not sufficiently described in the main text. We agree this commentary is needed for completeness. We have now added descriptive text to the Results section summarizing these findings:

**(1) Regarding overall numbers and events averted**: "When expanding eligibility to include the intermediate-risk group (≥7.5% risk), the risk-based approach identified 78.9 million adults (24.3%) and averted 3.04 million events (NNT=26) (**S3 Table**). The corresponding individualized benefit approach calibrated for similar event prevention (requiring iARR≥2.8%, "at least moderate-benefit") identified 76.7 million adults (23.6%) and averted 3.05 million events (NNT=25) (**S3 Table**)." (Page 14, Lines 180-186).

**(2) Regarding population overlap**: "For the at least intermediate-risk vs. at least moderate-benefit comparison, overlap increased to 76.5%, but the expanded risk-based approach still missed 9.3 million selected by the benefit approach (**Fig 3**)." (Page 16, Lines 221-224).

Additionally, as noted in the response, we confirm that for clarity and focus on our primary effectiveness metric (absolute number of CVD events averted), the column showing relative CVD risk reduction (%) has been removed from Table 1 in the revised manuscript.

**5. The authors state that "The conceptual diagram and detailed methods for evaluating these outcomes are provided in the S1 Appendix", but the conceptual diagram is shown in Figure 1.**

**Response:** We thank the Reviewer for catching this inconsistency regarding the location of the conceptual diagram. We have corrected the text in the Methods section. The revised sentence now accurately states: "Statin eligibility was assessed using two strategies: (i) the conventional absolute risk-based approach and (ii) the individualized benefit approach. Both required estimating baseline 10-year CVD risk and the expected iARR from statin treatment (Conceptual overview in Fig 1, details are provided in the S1 Appendix)." (Page 9, Line 66-70)

**General editorial requests**

(Note: not all will apply to your paper, but please check each item carefully)

*** We ask every co-author listed on the manuscript to fill in a contributing author statement, making sure to declare all competing interests. If any of the co-authors have not filled in the statement, we will remind them to do so when the paper is revised. If all statements are not completed in a timely fashion this could hold up the re-review process. If new competing interests are declared later in the revision process, this may also hold up the submission. Should there be a problem getting one of your co-authors to fill in a statement we will be in contact. Please do not add or remove authors without first discussing this with the handling editor. You can see our competing interests policy here:** [**http://journals.plos.org/plosmedicine/s/competing-interests**](http://journals.plos.org/plosmedicine/s/competing-interests)**.**

**Response:** We have added a section on *Author contribution* in the manuscript to state the contribution of each co-author clearly. In the *Competing interest* section, we stated, "I have read the journal's policy, and the authors of this manuscript have the following competing interests: Pei Gao reported receiving research funds from Bayer and Merck, but the funding sources had no relation to this study. All other authors of this study have reported that they have no relationships relevant to the contents of this paper to disclose.".

*** Please upload any figures associated with your paper as individual TIF or EPS files with 300dpi resolution at resubmission; please read our figure guidelines for more information on our requirements: http://journals.plos.org/plosmedicine/s/figures. While revising your submission, please upload your figure files to the PACE digital diagnostic tool, https://pacev2.apexcovantage.com/. PACE helps ensure that figures meet PLOS requirements. To use PACE, you must first register as a user. Then, login and navigate to the UPLOAD tab, where you will find detailed instructions on how to use the tool. If you encounter any issues or have any questions when using PACE, please email us at** [**PLOSMedicine@plos.org**](mailto:PLOSMedicine@plos.org)**.**

**Response:** We have uploaded all the figures associated with our paper (both in the manuscript and the Supporting information) as individual TIF.

*** Please ensure that the paper adheres to the PLOS Data Availability Policy (see http://journals.plos.org/plosmedicine/s/data-availability), which requires that all data underlying the study's findings be provided in a repository or as Supporting Information. For data residing with a third party, authors are required to provide instructions with contact information (web or email address) for obtaining the data. Please note that a study author cannot be the contact person for the data. PLOS journals do not allow statements supported by "data not shown" or "unpublished results." For such statements, authors must provide supporting data or cite public sources that include it.**

**Response:** In the *Data reporting* section, we have stated the source of our data: "The CHARLS is a public database; the data are available via the website https://charls.charlsdata.com/pages/Data/2015-charls-wave4/en.html.".

*** We expect all researchers with submissions to PLOS in which author-generated code underpins the findings in the manuscript to make all author-generated code available without restrictions upon publication of the work. In cases where code is central to the manuscript, we may require the code to be made available as a condition of publication. Authors are responsible for ensuring that the code is reusable and well documented. Please make any custom code available, either as part of your data deposition or as a supplementary file. Please add a sentence to your data availability statement regarding any code used in the study, e.g. "The code used in the analysis is available from Github [URL] and archived in Zenodo [DOI link]" Please review our guidelines at https://journals.plos.org/plosmedicine/s/materials-software-and-code-sharing and ensure that your code is shared in a way that follows best practice and facilitates reproducibility and reuse. Because Github depositions can be readily changed or deleted, we encourage you to make a permanent DOI'd copy (e.g. in Zenodo) and provide the URL.**

**Response:** We have added a sentence in the *Data reporting* section: "The code used in the analysis was adopted from the Prevention Impact and Efficiency (PIE) model: https://github.com/markpletcher/PIE-Model_Stata."

*** FINANCIAL DISCLOSURES: The funding statement should include: specific grant numbers, initials of authors who received each award, URLs to sponsors' websites. Also, please state whether any sponsors or funders (other than the named authors) played any role in study design, data collection and analysis, the decision to publish, or preparation of the manuscript. If they had no role in the research, include this sentence: "The funders had no role in study design, data collection and analysis, decision to publish, or preparation of the manuscript."**

**Response:** In the Financial Disclosure Statement section, we have stated that "This study was supported by the Noncommunicable Chronic Diseases - National Science and Technology Major Project (2024ZD0527406), the National Natural Science Foundation of China (82373662, 81973132) and the Beijing Natural Science Foundation (IS24047). Xun Tang received the Noncommunicable Chronic Diseases - National Science and Technology Major Project (2024ZD0527406), and the National Natural Science Foundation of China (82373662, 81973132), and Pei Gao received the Beijing Natural Science Foundation (IS24047). The URL of the Noncommunicable Chronic Diseases - National Science and Technology Major Project: https://service.most.gov.cn/. The URL of the National Natural Science Foundation of China: https://www.nsfc.gov.cn/english/site_1/index.html. The URL of the Beijing Natural Science Foundation: https://nsf.kw.beijing.gov.cn/bjnsfweb/. The study funders/sponsors had no role in the study design, data collection and analysis, decision to publish, or preparation of the manuscript.".

*** COMPETING INTEREST: All authors must declare their relevant competing interests per the PLOS policy, which can be seen here: https://journals.plos.org/plosmedicine/s/competing-interests
For authors with ties to industry, please indicate whether any of the interests has a financial stake in the results of the current study.**

**Response:** In the *Competing interest* section, we have stated that "I have read the journal's policy, and the authors of this manuscript have the following competing interests: Pei Gao reported receiving research funds from Bayer and Merck, but the funding sources had no relation to this study. All other authors of this study have reported that they have no relationships relevant to the contents of this paper to disclose.".

**FORMATTING - GENERAL
* Abstract: Please structure your abstract using the PLOS Medicine headings (Background, Methods and Findings, Conclusions). Please combine the Methods and Findings sections into one section.**

**Response:** We have complied with the journal's Submission Guidelines.

*** At this stage, we ask that you include a short, non-technical Author Summary of your research to make findings accessible to a wide audience that includes both scientists and non-scientists. The Author Summary should immediately follow the Abstract in your revised manuscript. This text is subject to editorial change and should be distinct from the scientific abstract. Ideally each sub-heading should contain 2-3 single sentence, concise bullet points containing the most salient points from your study. In the final bullet point of 'What Do These Findings Mean?', please include the main limitations of the study in non-technical language. Please see our author guidelines for more information: https://journals.plos.org/plosmedicine/s/revising-your-manuscript#loc-author-summary.**

**Response:** Following the author's guidelines, we have included an Author Summary following the Abstract.

*** Please express the main results with 95% CIs as well as p values. When reporting p values please report as p<0.001 and where higher as the exact p value p=0.002, for example. Throughout, suggest reporting statistical information as follows to improve clarity for the reader "22% (95% CI [13%,28%]; p</=)". Please be sure to define all numerical values at first use.**

**Response:** Revised as suggested.

*** Please include page numbers and line numbers in the manuscript file. Use continuous line numbers (do not restart the numbering on each page).**

**Response:** We have complied with the journal's Submission Guidelines.

*** Please cite the reference numbers in square brackets. Citations should precede punctuation.**

**Response:** We have complied with the journal's Submission Guidelines.

**FIGURES AND TABLES
* Please provide titles and legends for all figures and tables (including those in Supporting Information files).**

**Response:** We have complied with the journal's Submission Guidelines.

*** Please define all abbreviations used in each figure/table (including those in Supporting Information files).**

**Response:** We have complied with the journal's Submission Guidelines.

*** Please consider avoiding the use of red and green in order to make your figure more accessible to those with color blindness.**

**Response:** Revised as suggested.

**SUPPLEMENTARY MATERIAL
* Please note that supplementary material will be posted as supplied by the authors. Therefore, please amend it according to the relevant comments outlined here.**

**Response:** We have complied with the journal's Submission Guidelines.

*** Please cite your Supporting Information as outlined here: https://journals.plos.org/plosmedicine/s/supporting-information**

**Response:** We have complied with the journal's Submission Guidelines.

**REFERENCES
* PLOS uses the numbered citation (citation-sequence) method and first six authors, et al.**

**Response:** We have complied with the journal's Submission Guidelines.

*** Please ensure that journal name abbreviations match those found in the National Center for Biotechnology Information (NCBI) databases (http://www.ncbi.nlm.nih.gov/nlmcatalog/journals), and are appropriately formatted and capitalised.**

**Response:** We have complied with the journal's Submission Guidelines.

*** Where website addresses are cited, please include the complete URL and specify the date of access (e.g. [accessed: 12/06/2024]).

* Please also see https://journals.plos.org/plosmedicine/s/submission-guidelines#loc-references for further details on reference formatting.**

**Response:** We have complied with the journal's Submission Guidelines.

**STUDY TYPE-SPECIFIC REQUESTS

* Abstract: Please include the study design, population and setting, number of participants, years during which the study took place (enrollment and follow up), length of follow up, and main outcome measures.**

**Response:** We have complied with the journal's Submission Guidelines.

*** Please ensure that the study is reported according to the STROBE (or appropriate STOBE extension) guideline (available from: https://www.equator-network.org/reporting-guidelines/strobe) and include the completed STROBE (or STROBE extension) checklist as Supporting Information. Please add the following statement, or similar, to the Methods: "This study is reported as per the Strengthening the Reporting of Observational Studies in Epidemiology (STROBE) guideline (S1 Checklist)." When completing the checklist, please use section and paragraph numbers, rather than page numbers.**

**Response:** We have complied with the journal's Submission Guidelines.

*** For all observational studies, in the manuscript text, please indicate: (1) the specific hypotheses you intended to test, (2) the analytical methods by which you planned to test them, (3) the analyses you actually performed, and (4) when reported analyses differ from those that were planned, transparent explanations for differences that affect the reliability of the study's results. If a reported analysis was performed based on an interesting but unanticipated pattern in the data, please be clear that the analysis was data driven.**

**Response:** We have complied with the journal's Submission Guidelines.

*** Please state in the Methods section whether the study had a prospective protocol or analysis plan. If a prospective analysis plan (from your funding proposal, IRB or other ethics committee submission, study protocol, or other planning document written before analyzing the data) was used in designing the study, please include the relevant document(s) with your revised manuscript as a Supporting Information file to be published alongside your study and cite it in the Methods section. A legend for this file should be included at the end of your manuscript. If no such document exists, please make sure that the Methods section transparently describes when analyses were planned, and when/why any data-driven changes to analyses took place. Changes in the analysis, including those made in response to peer review comments, should be identified as such in the Methods section of the paper, with rationale.**

**Response:** We have provided our original statistical analysis and additional post hoc analyses were incorporated during the revision process in the Supporting information.

**Technical requests for the revision**

Journal Requirements:

**1) Please ensure your short title is correct in the submission form, including symbols, italicisation etc. Your short title will be typeset from the submission form directly. 
NOTE: Authors should provide a Short Title, around [70 characters] long, that states the topic of the study.**

**Response:** We have confirmed our short title as "Strategies of statin eligibility for primary prevention of CVD in Chinese".

**2) Thank you for stating "Pei Gao reported receiving research funds from Bayer and Merck, but the funding sources had no relation to this study. All other authors of this study have reported that they have no relationships relevant to the contents of this paper to disclose."**

**Please include a completed 'Competing Interests' statement in the text box when submitting your production task, including any COIs declared by your co-authors, written in full sentences.**

**If you have 'Competing Interests, please ensure the statement is in full sentences and includes the beginning statement: "I have read the journal's policy and the authors of this manuscript have the following competing interests:"
NOTE: If you have no competing interests to declare, please state "The authors have declared that no competing interests exist". You may also provide an updated statement via email.**

**Response:** Thank you for the guidance. We have now included a completed 'Competing Interests' statement in the text box when submitting our production task. The statement reads as follows: "I have read the journal's policy, and the authors of this manuscript have the following competing interests: Pei Gao reported receiving research funds from Bayer and Merck, but the funding sources had no relation to this study. All other authors of this study have reported that they have no relationships relevant to the contents of this paper to disclose." If any further modifications are required, please let us know.

**3) Thank you for stating "****Ethical approval for the CHARLS study was granted from the Peking University Institutional Review Board (IRB00001052-11015), all participants gave written informed consent before participation, and the blood-based biomarker collection study was also approved."

Please insert an Ethics Statement at the beginning of your Methods section, under a subheading 'Ethics Statement' and include the details
NOTE: It must include:

i) The name(s) of the Institutional Review Board(s) or Ethics Committee(s)
ii) The approval number(s), or a statement that approval was granted by the named board(s)
iii) A statement that formal consent was obtained (must state whether verbal/written) OR the reason consent was not obtained (e.g. anonymity). NOTE: If child participants, the statement must declare that formal consent was obtained from the parent/guardian.**

**Response:** In this round of revision, we have inserted an Ethics Statement at the beginning of the Methods section under the subheading 'Ethics Statement'. We have described the name of the Institutional Review Board, the approval number, and a statement that formal consent was obtained (written): "Ethical approval for the CHARLS study was granted from the Institutional Review Board at Peking University (IRB00001052-11015), all participants gave written informed consent before participation, and the blood-based biomarker collection study was also approved.^14^".

**4) Please ensure that the funders and grant numbers match between the Financial Disclosure field and the Funding Information tab in your submission form. Note that the funders must be provided in the same order in both places as well.
Currently, " Xun Tang received the National Natural Science Foundation of China (82373662,81973132) , and Pei Gao received the Beijing Natural Science Foundation (IS24047) )" are missing from the Funding Information**

**Response:** We have revised the text in the Financial Disclosure field and will modify the text in the Funding Information tab to ensure that the funders, grant numbers, and the funders are provided in the same order in both places: "This study was supported by the Noncommunicable Chronic Diseases - National Science and Technology Major Project (2024ZD0527406), the National Natural Science Foundation of China (82373662, 81973132) and the Beijing Natural Science Foundation (IS24047). Xun Tang received the Noncommunicable Chronic Diseases - National Science and Technology Major Project (2024ZD0527406), and the National Natural Science Foundation of China (82373662, 81973132), and Pei Gao received the Beijing Natural Science Foundation (IS24047). The URL of the noncommunicable chronic diseases - National Science and Technology Major Project is https://service.most.gov.cn/. The URL of the National Natural Science Foundation of China is: <https://www.nsfc.gov.cn/english/site_1/index.html>. The URL of the Beijing Natural Science Foundation is: <https://nsf.kw.beijing.gov.cn/bjnsfweb/>. The study funders/sponsors had no role in the study design, data collection and analysis, decision to publish, or preparation of the manuscript.".

**5) Please upload a copy of Figure [1 to 5] which you refer to in your text on the manuscript file. Or, if the figure is no longer to be included as part of the submission please remove all reference to it within the text.**

**Response:** We will upload a copy of all figures referred to in our text in the manuscript files when we resubmit our manuscript to the Editorial Manager.

**6) Please provide separate figure files in .tif or .eps format.For more information about figure files please see our guidelines:
https://journals.plos.org/plosmedicine/s/figures
https://journals.plos.org/plosmedicine/s/figures#loc-file-requirements**

**Response:** We will upload separate figure files in .tif of all figures in the main text and the appendix when we resubmit our manuscript to the Editorial Manager.

**7) Some material included in your submission may be copyrighted. According to PLOS's copyright policy, authors who use figures or other material (e.g., graphics, clipart, maps) from another author or copyright holder must demonstrate or obtain permission to publish this material under the Creative Commons Attribution 4.0 International (CC BY 4.0) License used by PLOS journals. Please closely review the details of PLOS's copyright requirements here: PLOS Licenses and Copyright. If you need to request permissions from a copyright holder, you may use PLOS's Copyright Content Permission form.

Please respond directly to this email and provide any known details concerning your material's license terms and permissions required for reuse, even if you have not yet obtained copyright permissions or are unsure of your material's copyright compatibility. Once you have responded and addressed all other outstanding technical requirements, you may resubmit your manuscript within Editorial Manager.

Potential Copyright Issues:

[Figure 4,5]: Please confirm whether you drew the images / clip-art within the figure panels by hand. If you did not draw the images, please provide (a) a link to the source of the images or icons and their license / terms of use; or (b) written permission from the copyright holder to publish the images or icons under our CC BY 4.0 license. Alternatively, you may replace the images with open source alternatives. See these open source resources you may use to replace images / clip-art:
- https://commons.wikimedia.org
- https://openclipart.org/**

**Response:** Thank you for the reminder. In the initially submitted figures, we manually drew Figures 4 and 5 (Fig 5 and Fig 6 in the new revision) in the Microsoft PowerPoint; the icons were added using PPT's built-in "Insert Icons" feature and inserted shapes such as rectangles via the "Insert Shapes" function. We have redrawn Figure 4 and Figure 5 by replacing the images or icons with open-source alternatives from https://commons.wikimedia.org and https://openclipart.org, as suggested by the editor. We will upload revised figures when we resubmit our manuscript to the Editorial Manager.
