# Supplementary material for: Absolute risk-based versus individualized benefit approaches for determining statin eligibility in primary prevention of cardiovascular diseases in Chinese populations: A modeling study
Source: PLoS Med. 2025 Jul 22;22(7):e1004556. doi: 10.1371/journal.pmed.1004556 (PMC12282892; doi:10.1371/journal.pmed.1004556)
Supplement: Attachment — Submitted filename: Response (R2) to reviewers.docx [file pmed.1004556.s020.docx]

Point-by-point Response to comments

Manuscript no.: PMEDICINE-D-25-00419

**Reviewer reports:**

**Reviewer #1: Thank you for revising the paper based on my comments. I enjoy reading the discussion about statin pathways and the new density plots on iARR.**

**Comment 1: I feel my comments are mostly addressed, even though I am still uncertain about the second line of the conclusion 'This suggests potentially improved efficiency in targeting individuals for statin therapy, which may be particularly relevant for shared decision-making in resource-constrained settings with distinct population risk profiles.' Efficiency arises from cost-effectiveness which has not been studied here. Given the population level effectiveness is similar I do not envision to see a massive increase in efficiency. Instead I would suggest to replace this line with 'The potential increase in population and individual level efficiency warrants further research'.**

**Response:** We appreciate the reviewer for this constructive suggestion. We agree that the term 'efficiency' in this context implies a formal cost-effectiveness analysis, which was not performed in our study. To avoid overstating our findings, we have removed it from the Conclusion as recommended. The sentence suggested by the reviewer is now added to the Limitation: "the potential for improved targeting efficiency at both population and individual levels warrants further research on cost-effectiveness." (Page 26, Line 436-438)

**Comment 2: In the first paragraph of the discussion 'was more efficient at the population level' does not seem to be supported by the findings. I'd also advise similar revision/rewording in the Conclusion section in the main text.**

**Response:** We thank the reviewer for this important point. We agree that since our primary analysis was calibrated to prevent a similar number of events at the population level, claiming superior “population-level efficiency” is not directly supported by the results. We have, therefore, revised the language in both the Discussion and the main Conclusion section. Specifically, we removed the claim and, in its place (Page 26, Line 436-438), aligning this section with the revised Abstract.

**Reviewer #2: Thank you. This version is the product of a major additional effort. But the product is impressive. This analysis now substantially extends understanding of the relative advantages and disadvantages of the Causal-Benefit Model of Cardiovascular Prevention versus the conventional Risk Model of Cardiovascular Prevention.**

**I am not requesting further change. My only comment for the authors to consider going forward is the issue of whether the benefit from statin therapy is attributable, at lease in part, to pleiotropic effects. I am not persuaded this is the case. Plaque stabilization could well be a happy outcome of lowering apoB particle number. But whether this is the case or not is irrelevant to the analysis. The concept is anchored in the CCT finding that reduction in event rate is close to constant per mmol/l reduction in LDL-C. This is a mathematic relation not a proof of mechanism.**

**Response:** We are grateful to the reviewer for the positive assessment of our revised manuscript and for the insightful comment regarding pleiotropic effects, which have helped us strengthen the framing of our assumptions and limitations. We agree that the cardiovascular benefit of statin therapy is largely attributable to LDL-C reduction, which is the central assumption of our modeling approach. The reduction in event rate is approximately constant per mmol/L reduction in LDL-C, as established in the Cholesterol Treatment Trialists' (CTT) Collaboration meta-analysis. This mathematical relationship, established by the CTT collaboration, is independent of the precise underlying biological mechanisms.

While our Limitations section addresses the modeling assumption of focusing on the LDL-C pathway, we believe the reviewer's perspective clarifies this point. Therefore, we have incorporated this message into the Discussion section by adding the following text:”Second, consistent with prior modeling studies [10,19,32,33], we assumed quantifiable benefit stems primarily from LDL-C reduction. While LDL-C lowering is the principal mechanism for statin benefit [34,35], this simplification overlooks other effects, such as plaque stabilization [36], and may underestimate benefits, particularly for high-risk individuals with lower LDL-C levels. However, our modeling approach is anchored in the well-established finding from the Cholesterol Treatment Trialists' (CTT) Collaboration that the reduction in cardiovascular event rates is approximately constant per mmol/L reduction in LDL-C [34]. This mathematical relationship enables robust estimation of expected benefits, irrespective of the underlying biological mechanisms. An alternative strategy, aligned with the principles of the Causal-Benefit model [11], is to apply a minimum benefit threshold to expand eligibility rather than only optimizing the selection of a fixed-size group. As demonstrated in our results, this strategy could substantially increase the number of cardiovascular events prevented by identifying a much larger number of eligible individuals." (Page 25-26, Line 416-432)

**Reviewer #3: The authors made appropriate changes to the manuscript and the responses to the comments were satisfactory.**

**No further comments.**

**Response:** We thank Reviewer #3 for the positive assessment and for confirming that our revisions have satisfactorily addressed the comments.

**Requests from Editors:**

**GENERAL
* Please confirm that your title complies with to PLOS Medicine's style. Your title must be nondeclarative and not a question. It should begin with main concept if possible. "Effect of" should be used only if causality can be inferred, i.e., for an RCT. Please place the study design ("A randomized controlled trial," "A retrospective study," "A modelling study," etc.) in the subtitle (i.e., after a colon).**

**Response:** We have revised the manuscript's title to conform to the journal's style. The study design has been added as "A modelling study" in the subtitle, following a colon.

*** Statistical reporting: Please revise throughout the manuscript, including tables and figures.**

**- Please report statistical information as follows to improve clarity for the reader ""22% (95% CI [13,28]; p</=)"".**

**- Please separate upper and lower bounds with commas instead of hyphens as the latter can be confused with reporting of negative values.
- Please repeat statistical definitions (HR, CI etc.) for each set of parentheses.**

**Response:** We have performed a comprehensive review of the manuscript and have revised all statistical reporting to align with the journal's guidelines. Specifically, we now use commas to separate confidence interval bounds, and define statistical abbreviations at each use within parentheses.

*** Please ensure that all abbreviations are defined at first use throughout the text (including statistical abbreviations). Please also check figures and tables.**

**Response:** We have carefully reviewed the entire manuscript (including main text, tables, and figures) and confirm that all abbreviations are defined upon their first use.

*** Please ensure that all tables and figures, including those in supplementary files, are appropriately referenced in the main text.**

**Response:** We confirm that all tables and figures, including those in the supplementary files, are cited sequentially within the main text.

*** The terms gender and sex are not interchangeable (as discussed in https://www.who.int/health-topics/gender#tab=tab_1 ); please use the appropriate term.**

**Response:** We have reviewed the manuscript and have used the term 'sex' to refer to biological attributes, in accordance with the journal's policy and WHO guidelines.

*** Please include the statement on code availability in the data availability statement (in the online submission form).**

**Response:** We confirm that a statement on code availability has been included in the Data Availability Statement as part of our revised submission.

*** In your study causality cannot be inferred. Please remove language that implies causality, such as impact. Please use associational language instead.**

**Response:** We thank the editors for this critical guidance. Recognizing that our modeling study does not establish causality, we have carefully revised the manuscript to replace words implying causation (e.g., 'impact') with more appropriate associational language. Key revisions include:

(1) In the Author Summary section

**Original:**

**The population impact** of the individualized benefit approach needed quantification in settings like China, where non-lipid factors (e.g., hypertension) significantly contribute to overall risk.

**Revision:**

**The potential population-level implications** of the individualized benefit approach needed quantification in settings like China, where non-lipid factors (e.g., hypertension) are key contributors to overall risk. (Page 5)

(2) In the Methods section

**Original:**

This allowed a direct comparison of population characteristics and efficiency under matched **population-level impact**.

**Revision:**

This allowed a direct comparison of population characteristics and efficiency under matched **population-level numbers of CVD events prevented**. (Page 12, Line 109-111)

(3) In the Discussion section

**Original:**

The major strength of this study is the use of nationally representative data from China, enabling us to assess **the impact** of various statin prevention strategies on a broad population.

**Revision:**

The major strength of this study is the use of nationally representative data from China, enabling us to assess **the numbers of statin eligibilities, the CVD events prevented, and the number needed to treat** for various statin prevention strategies in a broad population. (Page 24-25, Line 408-411)

(5) In the title of Table 1, Table 2, Table S3, Table S4, Table S6, Table S7, Table S8, Table S9

**Original:** "**Impact** and efficiency", for example, "Table 1. Impact and efficiency of the individualized benefit approach compared with treating high-risk group".

**Revision:** "Statin eligibilities, prevented CVD events, and efficiency", "Table 1. Statin eligibilities, prevented CVD events, and efficiency of the individualized benefit approach compared with treating high-risk group".

**ABSTRACT
* Please confirm that your abstract complies with our requirements, including providing all the information relevant to this study type https://journals.plos.org/plosmedicine/s/submission-guidelines#loc-abstract**

**Response:** We have reviewed the abstract following the PLOS Medicine guidelines for modeling studies and confirm that it meets the structural and content requirements.

*** Please ensure that all numbers presented in the abstract are present and identical to numbers presented in the main manuscript text.**

**Response:** We have performed a careful cross-check and confirm that all numerical data presented in the abstract are identical to those in the main manuscript.

*** When revising the Abstract, please include sufficient explanation and guidance throughout to ensure that the reader can understand and follow the synopsis of the results and conclusions.**

**Response:** In response to feedback from Reviewer #1 and the editors, we have revised the abstract to improve clarity. Specifically, we have now included key numerical results to better explain the differences in the iARR distributions between the two approaches. Furthermore, we have tempered the language in the conclusion to adopt a more cautious tone that is closely aligned with our findings, replacing the direct implication of improved efficiency. We also clarified how sampling weights were handled when reporting population counts, and more precisely described the overlap and divergence in statin eligibility between the two approaches.

*** We're not sure if the reader will understand when you write, "A total of 7,287 adults were analyzed, representing 324.6 million Chinese adults." Suggestion: "A total of 7,287 adults were analyzed, forming a cohort reflective of 324.6 million Chinese adults."**

**Response:** We thank the editors for this helpful suggestion. We have adopted the proposed wording to improve clarity and have revised the sentence in both the Abstract and the main Results section as "A total of 7,287 adults were analyzed, forming a cohort reflective of 324.6 million Chinese adults." (Page 3)

*** "The benefit approach uniquely identified 8.6 million, including 1.3 million with borderline risk (5%-7.5%)." – please explain in more detail. Suggestion: The benefit approach alone identified 8.6 million people who would highly benefit from statin therapy. This includes 1.3 million people with borderline risk (5%-7.5%), who would not be eligible for statin therapy under the absolute risk-based approach.**

**Response:** We appreciate the editors' request for clarification on this point. We have implemented this revision in the Abstract as: "The benefit approach alone identified 8.6 million people highly benefit from statin therapy, who would not be eligible for statin therapy under the absolute risk-based approach, and this includes 1.3 million people with borderline risk (5% to 7.5%)." (Page 3).

*** "The risk-based approach selected more individuals with low predicted iARR (longer lower-end tail in the iARR distribution), indicating better individual level targeting efficiency with the benefit approach." – We suggest including the numerical results here. Additionally, we believe the explanation from the rebuttal ("meaning it selects more individuals with low predicted benefit") would be useful.**

**Response:** Thank you for the suggestion. We have revised the abstract to include numerical data that quantifies the difference in the iARR distributions as “the risk-based approach selected more individuals with low predicted benefit (minimum iARR: 2.5% vs. 3.4%), resulting in a less efficient individual-level targeting profile (maximum iNNT: 41 vs. 29).” (Page 3). To align with the figure illustrating the distribution of iARR (Figure S2), we revised the reporting of outcome measures (e.g., in Table 1 and Table 2) to present the range of iARR (from the minimum to the maximum) instead of the interquartile range.

*** In the last sentence of the Abstract Methods and Findings section, please describe the main limitation(s) of the study's methodology.**

**Response:** As requested, we have added a sentence describing the study's main methodological limitation to the end of the Abstract's 'Methods and Findings' section as: " A key limitation of this study is that benefit was estimated primary from LDL-C reduction, which may neglect other biological mechanisms of statin effects and underestimate the total benefit." (Page 3).

**AUTHOR SUMMARY
* "Applying the Causal-Benefit model's minimum benefit threshold principle, the benefit approach recommended treatment for significantly more adults (46 million additional)…" – please check whether the use of the statistical term 'significant’ is supported by the data, and if not please remove it.**

**Response:** To avoid any potential misinterpretation as a claim of statistical significance, we have removed the word ‘significantly’ as requested.

**METHODS AND RESULTS
* “blood-based biomarker collection study was also approved [14].” – please provide the IRB approval number and clarify that written informed consent was provided for both studies.**

**Response:** Thank you for highlighting the need for this clarification. We have amended the Methods section to explicitly state the IRB approval number for the biomarker collection sub-study and to confirm that separate written informed consent was obtained, citing the original CHARLS biomarker publication (*Am J Epidemiol*. 2019;188:1871–1877). We have added the information: “The blood-based biomarker collection component of the study was also separately approved by the Institutional Review Board at Peking University (IRB00001052–11014) [14]. All participants gave written informed consent before participation.” (Page 9, Line 49-52)

*** l.58: When providing age, please ensure to include a unit, such as years. Please revise throughout the manuscript.**

**Response:** We have reviewed the manuscript and confirm that the unit 'years' has been added to all mentions of age for clarity.

*** Figure 2: Please clarify what the numbers in the circles represent and provide the denominator.**

**Response:** To clarify the data presented in Figure 2, we have added a detailed legend that explains the values within the Venn diagram as “Numbers outside the parentheses indicate the number of statin-treated individuals in each color-coded subgroup; numbers inside the parentheses indicate their proportion relative to the total statin-treated population of gaining at least a minimum benefit as the high-risk groups (iARR≥2.5%).”. The new legend clarifies that the numbers represent the population count for each subgroup, while the values in parentheses represent the proportion relative to total eligible population under the expanded benefit approach.

*** l.264, we suggest adding a qualifier: “This demonstrates the potential superior efficiency of the individualized benefit approach in excluding individuals predicted to derive minimal absolute benefit from statin therapy.”**

**Response:** Thank you for this suggestion. To better support this conclusion, we have strengthened the sentence by adding quantitative data describing the different iARR ranges for the two approaches, which provides direct evidence for the statement. The revised text incorporates the suggested qualifier and the new data to more robustly demonstrate the superior targeting efficiency of the benefit approach as: "The difference in iARR distribution tails was particularly evident in younger adults (aged 40-60 years): the lower bound of the iARR under the risk-based approach and the individualized benefit approach was 2.5% and 3.8%, respectively, and the maximum iNNT values were 41 and 26, correspondingly. (S2D Fig). This demonstrates the superior efficiency of the individualized benefit approach in excluding individuals predicted to derive minimal absolute benefit from statin therapy." (Page 19, Line 276-282)

*** Please check that any use of statistical terms (such as trend or significant) are supported by the data, and if not please remove them.**

**Response:** We have performed a careful review of the manuscript and have removed any uses of statistical terms, such as 'significant' or 'trend,' that were not supported by a corresponding p-value or formal statistical test.

*** Please specify the variables controlled for in all relevant Tables.**

**Response:** We appreciate this request for greater clarity. The outcomes in our tables (e.g., 'CVD events averted') are derived from a multivariable modeling process. To ensure transparency, we have added a legend to the relevant tables. This legend states that risk prediction was based on the 2019 WHO laboratory-based equations, which take into account age, sex, systolic blood pressure, total cholesterol, smoking status, and diabetes status. It also clarifies that the statin treatment effect was based on the CTT Collaboration meta-analysis, which inherently reflects the balanced characteristics of numerous RCTs.

*** S2 Figure: Please note that the graphs say “Individualize”.**

**Response:** We thank the editors for identifying this typo. We have corrected 'individualize' to 'individualized' in the S2 Figure.

**DISCUSSION
* Please remove all subheadings.**

**Response:** As requested, all subheadings within the Discussion section have been removed to create a single, continuous narrative.

**General editorial requests**

**1) We ask every co-author listed on the manuscript to fill in a contributing author statement. If any of the co-authors have not filled in the statement, we will remind them to do so when the paper is revised. If all statements are not completed in a timely fashion this could hold up the re-review process. Should there be a problem getting one of your co-authors to fill in a statement we will be in contact. YOU MUST NOT ADD OR REMOVE AUTHORS UNLESS YOU HAVE ALERTED THE EDITOR HANDLING THE MANUSCRIPT TO THE CHANGE AND THEY SPECIFICALLY HAVE AGREED TO IT.**

**Response:** We have provided the contributing author statement of all the co-authors in both our manuscripts and the online submission form.

**2) Please ensure that the paper adheres to the PLOS Data Availability Policy (see http://journals.plos.org/plosmedicine/s/data-availability), which requires that all data underlying the study's findings be provided in a repository or as Supporting Information. For data residing with a third party, authors are required to provide instructions with contact information for obtaining the data. PLOS journals do not allow statements supported by "data not shown" or "unpublished results." For such statements, authors must provide supporting data or cite public sources that include it.**

**Response:** We confirm that the manuscript adheres to the PLOS Data Availability Policy.

**3) Please review your reference list to ensure that it is complete and correct. If you have cited papers that have been retracted, please include the rationale for doing so in the manuscript text, or remove these references and replace them with relevant current references. Any changes to the reference list should be mentioned in the rebuttal letter that accompanies your revised manuscript.**

**Response:** We appreciate this important comment. We conducted a thorough check of all references cited in the manuscript and supplementary materials by systematically searching each reference in both PubMed and Web of Science. The reference list has been verified for accuracy and to ensure no retracted articles are cited. Accordingly, no changes to the reference list were required.
